# Potential of volatile organic compounds in the management of insect pests and diseases of food legumes: a comprehensive review

**DOI:** 10.3389/fpls.2024.1430863

**Published:** 2024-10-04

**Authors:** Leila Makhlouf, Karim El Fakhouri, Seid Ahmed Kemal, Ilyas Maafa, Issam Meftah Kadmiri, Mustapha El Bouhssini

**Affiliations:** ^1^ Laboratory of Entomology and Phytopathology, International Center for Agricultural Research in the Dry Areas (ICARDA), Rabat, Morocco; ^2^ AgroBioSciences Program, College of Agriculture and Environmental Sciences, Mohammed VI Polytechnic University, Ben Guerir, Morocco; ^3^ Plant and Microbial Biotechnology Center, Moroccan Foundation for Advanced Science, Innovation and Rescarch (MAScIR), Mohammed VI Polytechnic University, Ben Guerir, Morocco

**Keywords:** food legumes, volatile organic compounds, microbial VOCs, pest and disease management, cropping systems, crop breeding, pest and disease resistance

## Abstract

Cool season legumes (Faba bean, chickpea, lentil, pea, and grass pea) are important protein harvests for food and nutrition security in many countries. They play key roles in sustainable cereal production through their ecological benefits. However, diseases and pests attack continue to have a substantial impact on crop yield and quality. Although growers used different control options to manage these biotic stresses such as pesticide application, cultural practices, and resistant varieties, there is a pressing need for the development of new, more cost-effective and environmentally friendly solution to help farmers in facing the existing environmental issues. Recently, there is a growing interest among researchers in exploiting Volatile Organic Compounds (VOCs) for the elaboration of disease and pest control strategies in food legumes and other crops. These compounds have important functions in ecological relationships occurring between plants and their surrounding environment, as well as plants and others species, such as pests and pathogens. Due to their unique properties, VOCs can be employed in improving management alternatives for food legume diseases and pests. In this assessment, we investigated the role of VOCs in plant-pest and plant-pathogen interactions and their present applications in pest and diseases control strategies. We emphasized the ecological importance of employing plant VOCs in legume farming and crop breeding. Additionally, we highlighted the potential of microbial VOCs in facilitating microbe-microbe, microbe-plant and microbe-plant-pest interactions, along with their role in food legume protection.

## 1 Introduction

Cool-season food legumes such as faba bean, chickpea, lentil, field pea, and grass pea, play a vital role in food production, animal feed, and revenue generation in many regions (Semba et al., 2021). Globally, they occupy huge cultivated areas and are mainly grown for their edible protein-rich seed, and thus are named grain legumes (Calles, 2016).

In cropping systems that mostly rely on cereals, food legume crops are essential for enhancing soil fertility by fixing nitrogen. In Morocco, farmers practice wheat rotation with faba bean and benefit 48% over wheat monocropping (Yigezu et al., 2019).

However, in many regions of the world, their production exhibits significant yield fluctuations over the years due to abiotic and biotic factors. Pathogen invasions and insect pest infestations have damaging impact on both crop production and ecosystem worldwide. The surveillance and management of these biotic stresses are critical to increase crop yields and maintain food security for the expanding global population.

Farmers employ diverse strategies to cope with diseases and pests in food legume crops, involving agricultural approaches such as diverse crop rotation and adapting sowing schedules. Additionally, the planting of resistant varieties is a common approach (Sharma et al., 2016); however, its effectiveness is sometimes compromised by the emergence of new pathogen races and insect biotypes that can overcome the resistance in cultivated varieties.

Moreover, the application of fungicides and insecticides allow to reduce the pathogen and pest infestation, but their excessive use can induce resistance in target organisms, disrupt the soil’s microbial community, result in environment pollution with harmful chemicals (Li et al., 2012). Consequently, there is a need to develop new eco-friendly options to enhance pest management effectiveness (Haware and Nene, 1982; Brilli et al., 2019).

Over the recent decades, Volatile Organic Compounds produced by living beings have received growing attention in agricultural, environmental and ecological researches due to their various properties, and their potential applications in the biocontrol of plant pests and pathogens, as well as plant growth promotion (Choudhary et al., 2008; Pickett and Khan, 2016; Gualtieri et al., 2022; Devrnja et al., 2022; Russo et al., 2022; Patel et al., 2023).

Plants and microorganisms produce a wide range of Volatile Organic Compounds appertain to various chemical families with distinct biochemical origins like alcohols, aldehydes, aromatic compounds, esters, furans, monoterpenes, sesquiterpenes, hydrocarbons, and ketones (Kramer and Abraham, 2012). These secondary metabolic products typically possess low molecular weight (averaging below 300 Da), reduced boiling temperatures, and elevated vapor pressure (evaporating at 0.01 kPa around 20°C) (Morath et al., 2012). Consequently, they can diffuse into the air, ground, and fluids, exerting their effects over both short and long distances to ensure interactions between the organism and its environment, including connections among plants themselves (Delory et al., 2016; Sakurai et al., 2023), as well as exchanges between plant and pests or pathogens (Baldwin, 2010; Piechulla et al., 2017; Pérez-Hedo et al., 2024).

Due to their significant attributes and role as signaling compounds, VOCs provide several ecological and agricultural roles, and contribute to plant defense against pests and diseases (Piesik, 2011; Bezerra et al., 2021; Annaz et al., 2023).

This revise outlines the various applications of VOCs as natural and eco- friendly solution in food legume protection, highlighting their specific properties that make them effective as traps for managing different insect pest species. It indicates also the role of plant VOCs in inhibiting disease development, controlling insect pests and attracting natural enemies with a specific focus on their use in cropping systems. Furthermore, it demonstrates the exploitation of VOCs potential in plant breeding as non-invasive and rapid phenotypic tool for pest and disease resistance. Additionally, it details the different interactions mediated by microbial VOCs and their function in disease and pest management.

**Figure 1:**
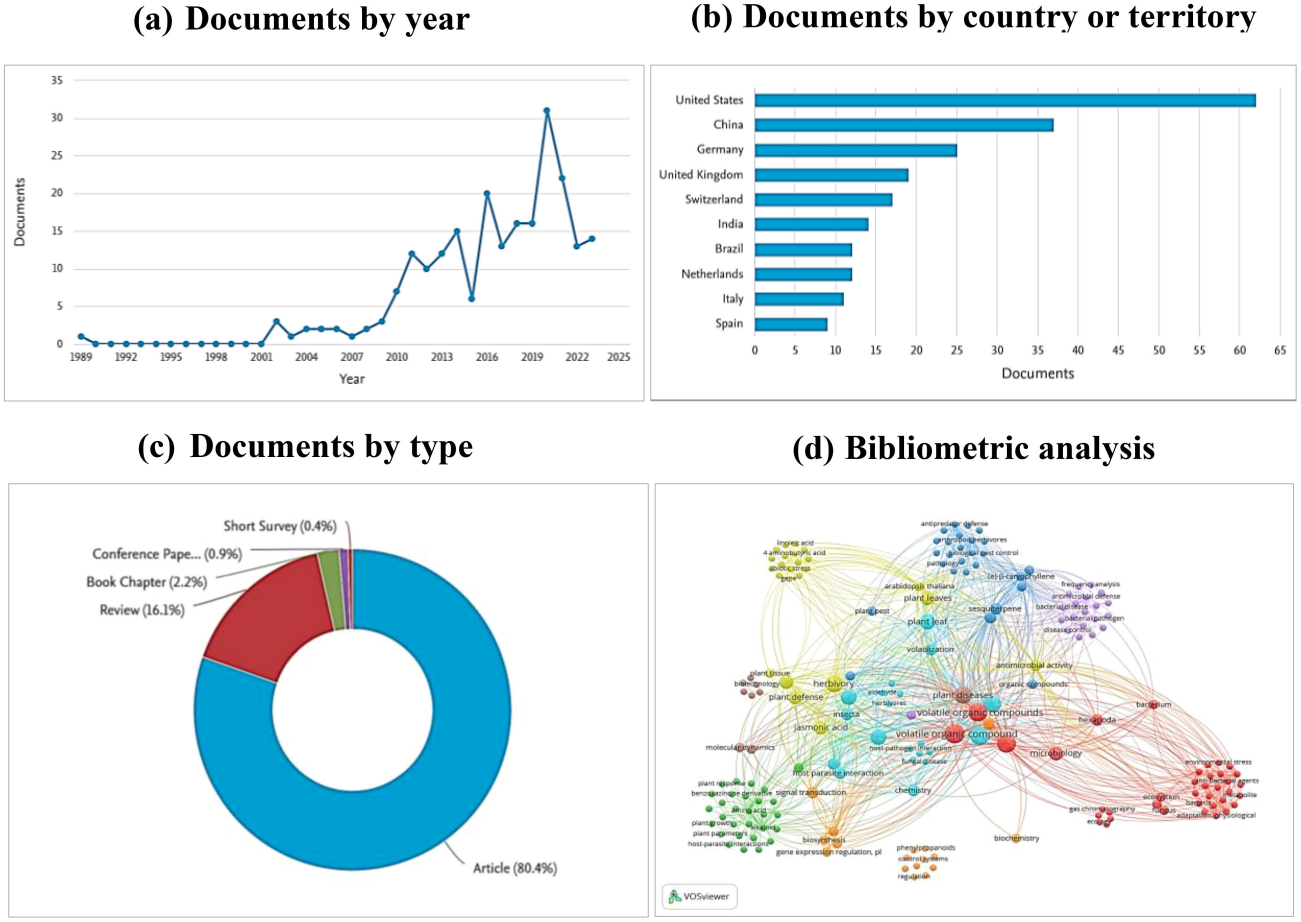
**(A)** the number of publications per year, **(B)** leading countries in publications on the role of VOCs in legume disease and pest management, **(C)** different type of documents in this search, **(D)** bibliometric network of keywords in publications on VOCs in disease and pest management of legume. From: VOSviewer.

This review utilized a bibliometric analysis to gather data from Scopus, Google Scholar, and Web of Science databases. The search was guided using the keywords “Volatile Organic Compounds AND food legume AND insect and disease management” resulting in a total of 224 documents. These documents comprised articles (80.4%), reviews (16.1%), book chapters (2.2%), conference papers (0.9%), and short surveys (0.4%) (**Figure 1C**).

Several bibliometric indices, including frequently used keywords were used to perform the Network analysis. This network was conducted using the VOS viewer processing software, revealing relationships among the identified keywords and offering a comprehensive overview of current use of Volatile Organic Compounds in legume crop protection (**Figure 1D**).

Furthermore, these findings facilitated an assessment of the significance of the published research. The classification of leading countries in article publications on the subject revealed that United States had the highest number of documents (62), followed by China with 37 documents and Germany with 25 documents (**Figure 1B**).

The publication trend indicated a substantial increase in the number of articles from 2015 to 2020, a slight decrease between 2020 and 2021, and a subsequent rise between 2021and 2023, indicating a growing interest in the topic (**Figure 1A**).

## 2 Current progress in using plant VOCs for pest and disease management in food legume crops

Plants release a diverse set of volatile organic compounds (VOC), either naturally or as reaction to both biotic and abiotic stressors, to cope with pest attack or pathogen invasion (Maffei et al., 2007; Dicke and Loreto, 2010; Mutyambai et al., 2016; Castro et al., 2017; Karolkowski et al., 2021).

**Figure 2:**
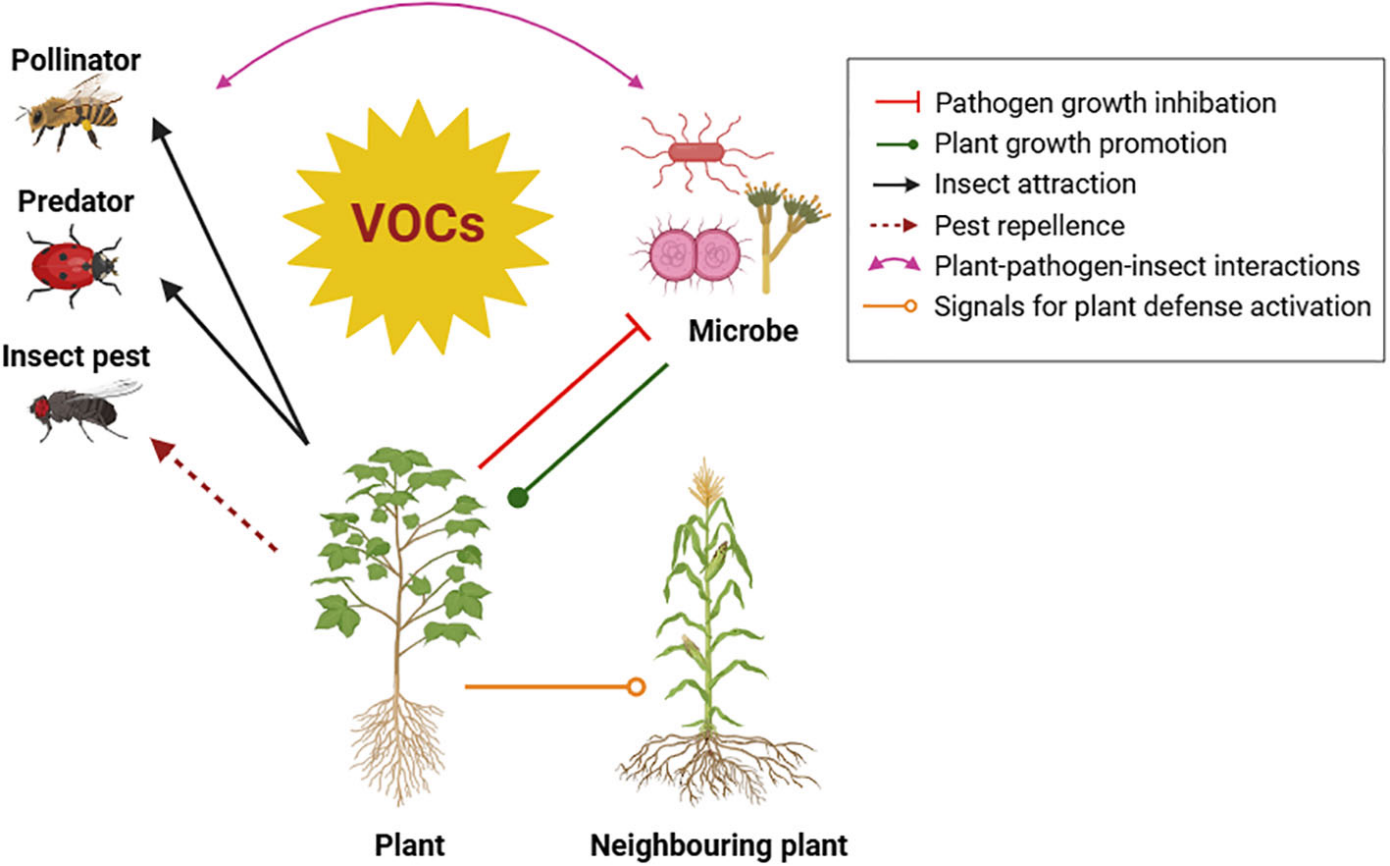
Diverse roles of Volatile Organic Compounds (VOCs) in mediating plant interactions with the environment.

These chemical signals serve multiple purposes for plants, including defense against pests and diseases, attraction of pollinators and other beneficial organisms, and communication with neighbouring plants (**Figure 2**) (Annaz et al., 2023; Ficke et al., 2021). An increasing attention is emerged for reducing reliance on chemical pesticides by adopting new sustainable, natural, and eco-friendly solution for effective pest and disease control like exploiting the ecological and agronomic potential of VOCs to protect plants (Brilli et al., 2019).

The following briefly outlines the functions of VOCs in pest and disease management and other ecological services.

### 2.1 VOCs and pest management

Due to their attractive or repellent action, VOCs can influence the feeding behavior of insect pests, their reproduction, their olfactory perception, and the selection of their hosts (Hegde et al., 2011; Santos et al., 2015; Bruno et al., 2018; Singh et al., 2021). Therefore, they can be used as pest traps to control insect population dynamics.

Understanding and deciphering the chemical ecology of insects may offer opportunities for enhancing eco-friendly methods to fight against bio-aggressors. This involves the exploitation of repellent or disruptive volatile organic compounds (VOCs) to annoy harmful insects, as well as the use of attractive compounds for pest trapping (Reisenman et al., 2016; Fountain et al., 2017; Wyatt, 2018) (**Table 1**).

**Table 1:** Diverse roles of Volatile Organic Compounds in plant-pest interactions.

	Insect pest	Plant emitting VOCs	Role of VOCs in plant-pest interraction
Leafminers	Serpentine leafminer, *Liriomyza huidobrensis*	Pea	Influence reproduction (attraction of male and female flies) (Ge et al., 2019)
	Vegetable leafminer, *Liriomyza sativae*	Faba bean	Attraction of parasitoid *Diglyphus isaea* (Zhao and Kang, 2002)
	Chickpea leafminer, *Liriomyza cicerina*	Chickpea	Deterrent and toxic potential (a means of resistance against insects) (Soltani et al., 2020)
Aphid	Black bean aphid, *Aphis fabae*	Faba bean	Influence feeding behaviour by reducing the time spent by the aphid on its host (Webster et al., 2008)
Beetle	Bean seed beetle, *Bruchus rufimanus*	Faba bean	Influence the olfactometry perception of female *B. rufimanus* (Bruce et al., 2011)
Weevils	Sitona weevil (PLW), *Sitona lineatus*	Pea ana Faba bean	Attraction of weevil in combination with an aggregation pheromone (Onge et al., 2018)
	The pea weevil (*Bruchus pisorum* L.)	Pea	Serve as inherent cues guiding *B. pisorum* male and female in locating suitable hosts (Ceballos et al., 2015)
Bean bug	Bean bug, *Riptortus pedestris*	Soybean	Interact synergistically with the aggregation pheromone to attract the bean bugs (Song et al., 2022)
Stink bug	Southern green stink bug, *Nezara viridula*	Faba bean	Affect the egg parasitoid *Trissolcus basalis* attraction (influence their host selection) (Tariq et al., 2013)
Pod borers	Legume pod borer, *Maruca vitrata*	Cowpea	Influence olfactory behavior response of female moths and the selection of oviposition site (Zhou et al., 2015)
	The gram pod borer, *Helicoverpa armigera*	Chickpea	Enhance the foraging activity of *Trichogramma* spp (Pawar et al., 2023)
	Faba bean stem borer, *Lixus algirus*	Faba bean	Attract both male and female *L. algirus* (Ait Taadaouit et al., 2021a)
Storage insect pest	The adzuki bean weevil, *Callosobruchus chinensis*	Clove, holy basil, lemongrass, turmeric	Oviposition deterrence, antifeedant activity, F1 progeny inhibition, and adult repellent activity (Mario et al., 2023)
	Cowpea weevil, *Callosobruchus maculatus*	Grass pea	Host locating (Adhikary et al., 2015)

#### 2.1.1 Legume leaf miner

The leaf miner, *Liriomyza spp* (Diptera: Agromyzidae), is among the most threatening insect pests to chickpea crop (*Cicer arietinum* L.) and other legumes in the Mediterranean region (Chrigui et al., 2020; Sabraoui et al., 2019). Adult leaf miners do not possess pheromone specific to their species; they utilize herbivore-induced plant volatiles HIPVs triggered by female punctures, which include green leaf volatile (GLVs), terpenoids and oximes, as cues for the host plant location. Also, natural enemies can exploit these HIPVs to locate their hosts.

**Figure 3:**
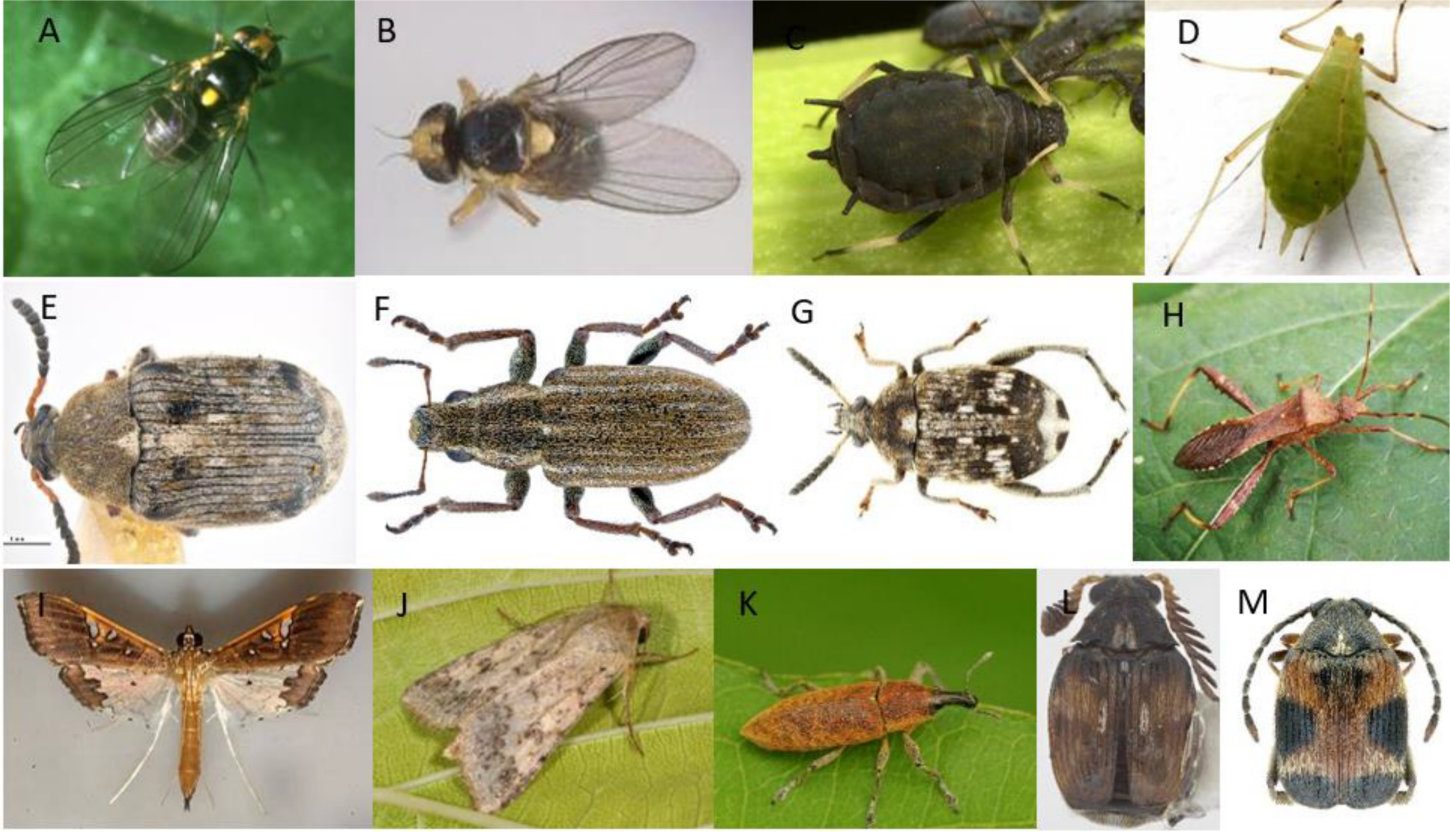
**(A)** Liriomyza huidobrensis; **(B)** Liriomyza sativae; **(C)** Aphis fabae; **(D)** Acyrthosiphon pisum; **(E)** Bruchus rufimanus; **(F)** Sitona lineatus L.; **(G)** Bruchus pisorum L.; **(H)** Riptortus pedestris; **(I)** Maruca vitrata **(J)** Helicoverpa armigera; **(K)** Lixus algirus; **(L)** Callosobruchus chinensis; **(M)** Callosobruchus maculatus.

For example, in pea plant, HIPVs such as (Z)-3-hexenyl-acetate and (Z)-3-hexenol promote the attraction of male and female flies of the pea leafminer *Liriomyza huidobrensis* (**Figure 3A**) and mating occurs on host leaves (Ge et al., 2019).

Moreover, bean plant odors such as cis-3-hexen-1-ol and 4-hydroxy-4- methyl-2-pentanone, produced by healthy and damaged bean plants, are reported in attracting leafminer (*L. sativae*) (**Figure 3B**) and its parasitoids *Diglyphus isaea* (Zhao and Kang, 2002). So these herbivores induced volatiles (HIVs) can be utilized to trap the pest or to attract its natural enemy.

#### 2.1.2 Aphids and virus on legumes

Legumes such as faba beans, chickpeas, lentils and peas are susceptible to many viruses of which 42 are identified as transmitted by vectors, mainly by insects (Makkouk, 2020; Jones and Roger, 2021; Adane and Vetten, 2022; Tatineni and Hein, 2023). The major vectors for legume viruses are aphids, leafhoppers, thrips, and beetles (Guerrieri and Digilio, 2008; Congdon et al., 2017).

For example, the black bean aphid, *Aphis fabae* (Hemiptera: Aphididae) (**Figure 3C**) poses a significant threat to faba beans (*Vicia faba)*, inflicting harm by feeding and also by transmitting plant viruses.

The aphids is attracted to its host through a mixture of volatile compounds released by *V.faba* plant, which consists of (Z)-3-hexen-1-yl acetate, 1-hexanol, (Z)-3-hexen-1-ol, benzaldehyde, (E)-2-hexenal, 6-methyl-5-hepten-2-one, octanal, (R)-(-)- linalool, methyl salicylate, decanal, undecanal, (E)-β-farnesene, (E)-β-caryophyllene, (S)-(-)-germacrene D and (E,E)-4,8,12-trimethyl1,3,7,11-tridecatetraene (TMTT).

Among this blend, the molecules octanal, (R)-(-)-linalool and (S)-(-)-germacrene D can reduce the duration that aphid remains on its host. However, the presence of these compounds in VOCs blend has no effect on the aphids’ behavior which underlining the importance of the blend effect on aphid behavior over individual compounds’ effect (Webster et al., 2008).

The pea aphid, *Acyrthosiphon pisum* (Hemiptera: Aphididae) (Harris), (**Figure 3D**) can consume its host plant, *Vicia faba*, without causing alterations in plant VOCs release. The volatile organic compound amount emitted by affected bored beans stayed consistent or reduced compared to undamaged plants. For example, aphid feeding decreased the emission of sesquiterpene (E)-ß-ocimene and GLV (Z)-3-hexenyl acetate in comparison with intact plants. Contrarily, the GLV (E)-2-hexenal emission is not impacted by aphid attack.

Also, Pea aphid can prevent the emission of some VOCs during feeding, such as ß-caryophyllene, (E)-ß-ocimene and (E, E)-4,8,12-trimethyl-1,3,7,11-tridecatetraene (TMTT), which can attract its parasitoid, *Aphidius ervi*. So the suppression of some VOCs production by aphid feeding make the plant incapable to attract natural enemies and protect itself (Schwartzberg et al., 2011).

#### 2.1.3 Legume beetle and weevil

The bean seed beetle, *Bruchus rufimanus* (Coleoptera: Chrysomelidae) (**Figure 3E**) presents a significant economic threat to beans, mainly field beans (*Vicia faba*) sown during the spring and winter seasons (Carrillo-Perdomo et al., 2018).

Bruce et al. (2011) showcased the significance of plant host-emitted volatile organic compounds in monitoring *B. rufimanus*. Nine active compounds were identified from *V. faba* cv. ‘Sutton dwarf’ flowers, including cinnamyl alcohol, cinnamaldehyde, myrcene, (R)-limonene, (E)-ocimene, (R)-linalool, 4-allylanisole, (E)-caryophyllene, and α-humulene.

The semiochemicals derived from host plant flowers and male bruchids induced electrophysiological and behavioral reactions in female *B. rufimanus*, which may be utilized to attract and trap the pests in the field (Dell'Aglio and Tayeh, 2023).

Although host plant odors are attractive as a blend, their individual exposure to insects may be less effective and even repellent. The proportion of each compound in the VOCs blend that the host plant naturally released is critical for communicating with insects. Therefore, to create an effective attractant, it’s crucial to mimic these natural ratios accurately.

In olfactometer bioassays, the chosen headspace collections and synthesized molecules were evaluated, and then incorporated into semiochemical lures for traps. During field trials, cone baits loaded with a combination of three floral volatile compounds, including (R)-linalool, cinnamyl alcohol, and cinnamaldehyde, trapped significantly more male and female of *B. rufimanus* compared to unbaited control traps.

Sitona weevil (PLW), *Sitona lineatus* L., (Coleoptera: Curculionidae) (**Figure 3F**) is a pest of pea (*Pisum sativum* L.), faba bean (*Vicia faba* L.) and other legume crops. Larvae that feed on root nodules reduce nitrogen fixation and cause the most damage. This pest could be managed by using an aggregation pheromone 4-methyl-3,5-heptanedione, in combination with host plant volatiles such as linalool, (Z)-3-hexenol, and (Z)-3-hexenyl acetate. The addition of plant volatiles to the aggregation pheromone can increase weevil attraction, however plant volatiles solely didn’t attract *S. lineatus* adults (Onge et al., 2018).

The pea weevil (*Bruchus pisorum* L.) (Coleoptera: Bruchidae) (**Figure 3G**) stands among the most damaging pests of peas (*Pisum sativum* L.) (Mendesil et al., 2016; Reddy et al., 2018; Aznar-Fernández and Rubiales, 2019). Ceballos et al. (2015) explored how the volatiles emitted by pea plants can affect the electrophysiological and behavioral reactions of *B. pisorum*, employing electroantennography (EAG) and olfactometry tests.

Volatiles produced at various developmental phases were extracted in headspace by Porapak Q tubes and identified using gas chromatography coupled to mass spectrometry (GC-MS) (Makhlouf et al., 2024). The Analysis using GC-MS showed variations, both qualitative and quantitative, in plant-emitted volatiles across different phenological stages. Terpenes were the most frequent compounds in every stage, with terpinene and 1-S-verbenone exclusively detected during the flower stage. Flowers released large amounts of all compounds, excluding myrcene and n-dodecane. Pea pods released low amount of (Z)-2-hexen-1-ol, 2,4-hexadienal, α-pinene, β-pinene, myrcene, and limonene, except for n-dodecane that was prevalent at this phase.

The highest concentration of compounds coincided with the vegetative and flower stages, consistent with Dudareva et al.’s (2004) findings, indicating that volatiles emission intensified when leaves are young and flowers are ready for pollination.

In olfactometer bioassays, volatiles produced in all growth phases prompted an attractant behavioral reaction from both male and female *B. pisorum*. Significantly, the female *B. pisorum* displayed a grater attraction to pod volatiles than other phenological stages. These volatiles released by the flowers and pods of pea plants act as natural signals directing *B. pisorum* to find appropriate hosts.

#### 2.1.4 The bean bug and stink bug

The bean bug *Riptortus pedestris* (Fabricius) (Heteroptera: Alydidae) (**Figure 3H**), is a generalist pest that mainly attack legumes particularly soybean, can recognize its host through the plant’s volatile organic compounds (VOCs).

Research carried out by Song et al. (2022) revealed that both sexes of *R. pedestris* are able to perceive volatiles emitted from soybeans such as (*Z*)-3-hexen-1-ol, (*Z*)-3-hexenyl acetate, 4-ethylbenzaldehyde, α-farnesene, and methyl salicylate. When assessed in controlled laboratory settings, bean bugs adult showed no specific behavioral responses to single molecule; however, they did demonstrate strong preference towards a specific combination of synthetic volatile compounds of soybean.

In natural conditions, the mixture of soybean volatiles didn’t considerably lure *R. pedestris*. However, it showed a synergistic interaction with the aggregation pheromone, effectively attracting the bean bugs. These findings emphasize the importance of host plant volatiles in the perceptive behavior of the bean bug, shedding light on the colonization dynamics of *R. pedestris* in soybean plantations.

#### 2.1.5 Legume pod borer

Zhou et al. (2015) have investigated how the female *Maruca vitrata* (Lepidoptera: *Crambidae*) (**Figure 3I**), a major pest in cowpea cultivation, responds to the volatiles emitted from *Vigna unguiculata* flowers and their role in the selection of host plants.

By employing gas chromatography coupled to electroantennography (GC-EAD) and gas chromatography-mass spectrometry (GC-MS) analysis, 17 major volatile compounds produced by *V. unguiculata* have been determine, involving butyl ester, butanoic acid, limonene, butanoic acid octyl ester, 4-ethylpropiophenone, 1H-indol-4-ol, and 2-methyl-3-phenylpropan.

During the field trials, six compounds from these floral volatiles successfully lured female moths and revealed notable distinctions in comparison with the control bait. These findings indicated that cowpea VOCs likely influence the scent-driven behavioral reaction of female moths, which in turn affecting their choice of egg-laying locations. This insight offers valuable understanding for investigating monitoring effectiveness and combined pest control approaches against the legume pod borer in agricultural settings.

The chickpea pod borer, *Helicoverpa armigera* (Lepidoptera: Noctuidae) (**Figure 3J**) presents a significant threat to chickpea crops. It can tunnel into the pods during the reproductive stage which resulting in major decreases in productivity (El Fakhouri et al., 2022; Boulamtat et al., 2019). *Trichogramma* spp., egg parasitoid, is used as biological control agents against Lepidoptera pests. Plant volatiles such as n-octadecanoic acid, n-hexadecanoic acid, and octadecane have been proven to improve the foraging function of *Trichogramma spp*, mainly octadecane which has been detected in several plant volatile profiles and is known to be attractive to *Trichogramma* spp. Pawar et al. (2023) demonstrated that applying a kairomone gel formulation of octadecane (Saturated hydrocarbon) after 24h of the *Trichogramma chilonis* release in chickpeas have improved the biological control capability of *T. chilonis* towards *H. armigera* larvae, leading to decreased pod damage and an increased chickpea grain yield.

Faba bean stem borer, *Lixus algirus* L. (Coleoptera: Curculionoidae) (**Figure 3K**), is regarded as one of the main insect pests affecting faba beans in the Mediterranean area (Ait Taadaouit et al., 2021a, b). Their extensive damage is primarily attributed to larval feeding within the plant stems. El Fakhouri et al. (2021) conducted experiments using both small wind tunnels and olfactometer bioassays, revealing that the volatiles emitted by healthy host plants during the flowering stage attract both male and female *L. algirus* significantly. The study also demonstrated that faba bean plants exhibit distinct volatile profiles based on the degree of infection, and the growth stage (VOCs of vegetative stage plants are different from those of the blooming stage). During the flowering stage of the infested plants, sixty-six compounds with notable GLV amounts such as 3-Hexen-1-ol, acetate, (Z)-, 1-Hexanol, 2-ethyl-, 3-Hexen-1-ol, 1 acetate, (E)- were released.

#### 2.1.6 Storage pests

Storage losses caused by insect pests in food legumes contribute significantly to food and nutrition insecurity, leading to reduced incomes for growers. Smallholder farmers, relying on traditional storage methods, often experience substantial losses, surpassing 70%. Reports indicate storage damages reaching up to 50% in certain essential legume crops including chickpea, faba bean, lentil, and pea (Keneni et al., 2011). Smallholder farmers employ various pest management strategies, including insecticide seed treatment, the use of botanicals, the adoption of improved storage structures and bags, and fumigation, which is the most common method for grain protection against insect pests. However, the two primary fumigants methyl bromide and phosphine, have encountered challenges due to environmental concerns such as decreasing ozone levels and biological factors like insect resistance, which highlight the need for urgent development of eco-friendly alternatives (Rajendran and Sriranjini, 2007). Volatile organic compounds emitted from different plants serve as an efficient tool for the control of many storage pests (Singh et al., 2021).

The adzuki bean weevil, *Callosobruchus chinensis* (L.) (Coleoptera: Chrysomelidae) (**Figure 3L**) is a highly harmful insect of stored legumes due to its highly destructive nature. In a study conducted by Mario et al. (2023), the application of plant powder fumigants emerged as a sustainable and economical strategy for managing pests of stored product. The effectiveness of powder fumigants of four plants including clove, holy basil, lemongrass, and turmeric was assessed. These plants exhibited substantial efficacy, causing mortality rates of up to 100% in *C. chinensis*. Additionally, they demonstrated oviposition deterrence, antifeedant activity, suppression of F1 offspring, and repellent efficacy for adults. Consequently, this multifaceted impact resulted in a reduced percentage of bean damage and weight loss.

Examining of the volatile organic compounds found in the four plant powder fumigants revealed key constituents responsible for their anti-pest properties. Clove was characterized by eugenol and caryophyllene, holy basil by estragole, lemongrass by α-Citral and β-Citral, and turmeric by α-zingiberene and β-sesquiphellandrene. Notably, the powder fumigant from clove plant has proved outstanding effectiveness across all observed variables, displaying remarkable bioefficacy despite the minimal quantity applied.

*Callosobruchus maculatus* (F.) (Coleoptera: Bruchidae) (**Figure 3M**) is a threatening stored grain pest affecting *Lathyrus sativus* L. (Leguminosae), widely referred to as khesari, in India, Bangladesh, and Ethiopia. This pest has the ability to locate its host by detecting the volatile organic compounds released from the host seeds (Hamdi et al., 2017).

A total of 23 volatiles were identified and quantified in different types of healthy khesari seeds including Bio L 212 Ratan, Nirmal B-1, WBK-13-1, and WBK-14-7, using gas chromatography coupled to mass spectrometry and gas chromatography-flame ionization detector analyses. Nonanal emerged as the predominant compound in the seeds of the two varieties Bio L 212 Ratan and WBK-13-1, then we found farnesyl acetone. Conversely, Nirmal B-1 and WBK-14-7 khesari seeds were characterized by farnesyl acetone as the predominant volatile, followed by nonanal.

The Y-shaped glass tube olfactometer bioassays were used to study the olfactory reactions of female *C. maculatus* towards volatile blend released by different varieties of khesari seed, as well as distinct synthetic compounds, and their mixture. The results indicated that this pest preferred the entire volatile mixture emitted by Bio L 212 Ratan seeds compared to those from the other three types. *C. maculatus* was attracted to five different synthetic compounds including 3-octanone, linalool oxide, 3-octanol, 1-octanol, and nonanal.

Notably, a synthesized mixture consisting of linalool oxide, 3-octanone, 3-octanol, 1-octanol, and nonanal at concentrations of 448, 390, 1182, 659, and 8114 ng/20 μl methylene chloride, respectively, proved to be the greater appealing to *C. maculatus*. This mix holds promise for potential use in *C. maculatus* pest control programs, like lured traps (Adhikary et al., 2015).

Furthermore, Ajayi et al. (2015) have investigated the host selectivity of *C. maculatus* female regarding seeds from three legume cultivars, Ife-brown and black-eyed cowpeas (*Vigna unguiculata* L.) and soybean (*Glycine max* L.). Results revealed that *C. maculatus* selects its host based on the odor and 2-ethyl hexanol has been identified as a possible attractant for this pest, which is more produced in Ife-brown cowpea.

### 2.2 VOCs and disease management

Plant overcomes pathogen attack by producing a set of volatile organic compounds (VOC) from different organs including seeds, leaves, roots, and nodules (Singh et al., 2022).

These VOCs serve as chemical signals that mediate interactions between plant and pathogen by reducing disease development, as well as, plant and their neighbouring plants by inducing resistance against pathogen attack (Kasote et al., 2023; Kong et al., 2024).

#### 2.2.1 VOCs in plant-pathogen interaction

**Figure 4:**
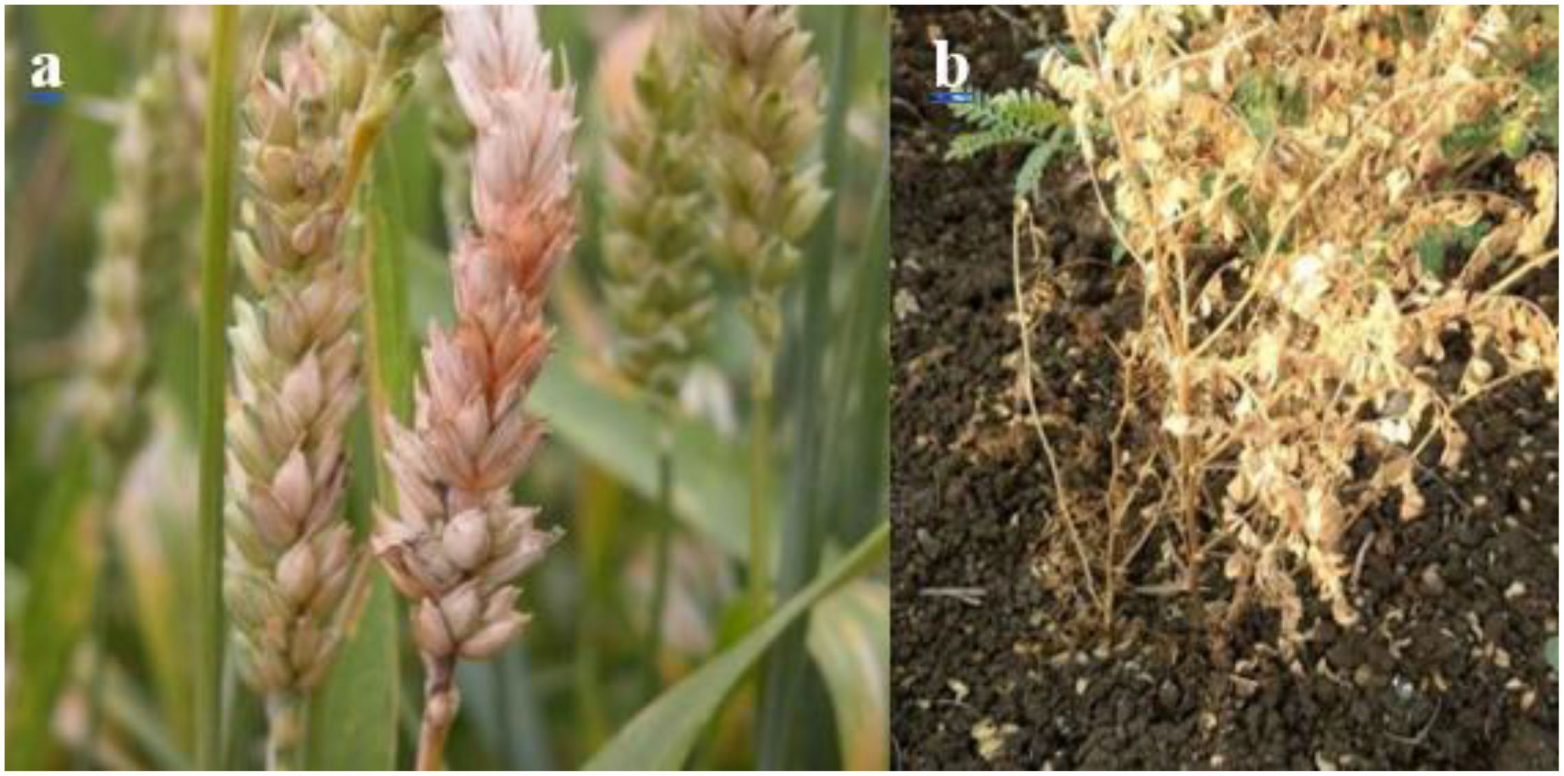
Fusarium symptoms **(A)** Fusarium head blight (FHB), caused by *Fusarium graminearum*, **(B)** Fusarium wilt on chickpea caused by *Fusarium oxysporum (f)* sp. *Ciceris* (InsectImages, 2018).

Fusarium species that are associated with chickpea plants are considered one of the most serious biotic stresses that affect chickpeas in the main growing regions (Lokesh et al., 2020; Sampaio et al., 2020). Chickpea plants can defend themselves against *Fusarium* attacks (**Figure 4**) by producing different volatile organic compounds (**Table 2**). Research conducted by Cruz et al. (2012) showed that chickpea VOCs, such as 1-hexanol, trans-2-hexenal, 1-penten-3-ol, cis-3- hexen-1-ol, and trans-2-hexen-1-ol, have negatively impacted both *Fusarium avenaceum* and *F. graminearum* development, however the intensity of their impact differed. For instance, trans-2-hexenal and trans-2-hexen-1-ol were highly effective, fully inhibiting *F. avenaceum* development even at the minimum dosage (2µl). Every VOCs have totally suppressed the pathogen growth at the highest dosage (50 µL), except for 1-penten-3-ol that decreased development by about 50% in comparison with the control without VOCs.

**Table 2:** Major volatile organic compounds of chickpea leaves.

Chemical family	Name of compound
**Aliphatic Alcohols**	Ethanol
	1-penten-3-ol
	1-hexanol
	Cis-3-hexen-1-ol
	Trans-2-hexen-1-ol
	3-methyl-1-butanol
**Aliphatic Aldehydes**	Trans-2-hexenal
**Aliphatic Ketones**	3-hydroxy-2-butanone
**Aromatic compounds**	3-methylbenzaldehyde
	Naphthalene

Bioassays performed in Petri plates revealed that volatile organic compounds (VOCs) produced by chickpeas, when tested individually, exhibited greater efficacy toward *F. graminearum* and *F. avenaceum* compared to VOCs produced by wheat (Cruz et al., 2012).

However, VOCs released from plants can exhibit harmful effects if they are found in high concentrations. For example, 2-ethyl-1-hexanol produced by chickpea may have an adverse effect on plant growth and also can prevent *Fusarium* development (Horiuchi et al., 2007; Barney et al., 2009).

**Figure 5:**
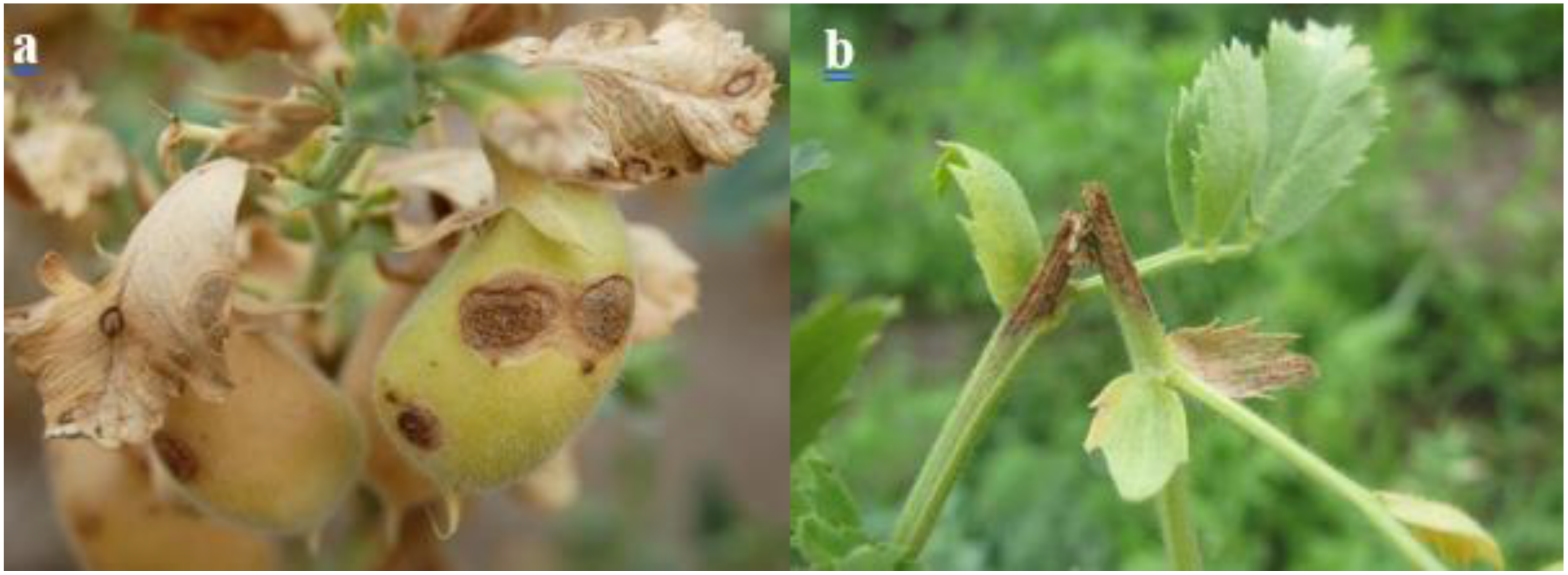
Ascochyta blight symptoms on chickpea plant **(A)** Brown lesions on leaves and pods, **(B)** Breakage of the stem (InsectImages, 2018).

Additionally, Ascochyta blight, caused by *Ascochyta rabiei* (Class: Coelomycetes, order: Sphaeropsidales) (Telomorph: *Didymella rabiei*; class: Dothideomycetes, order: Pleosporales) (Krimi et al., 2022) (**Figure 5**), stands as the major devastating foliar disease that affects chickpea plant (Gaur et al., 2012). It represents a serious obstacle to winter chickpea production (Houasli et al., 2021). To protect themselves from *A. rabiei* invasion, chickpea plants release a set of VOCs that can affect the pathogen’s development. According to Cruz et al. (2012), the release of the two volatile compounds 1-penten-3-ol and cis-3-hexen-1-ol, was triggered by the *A. rabiei* fungus. Besides, chickpea VOCs production was related to Ascochyta blight severity. However, research carried out by Oliva et al. (1999) has shown that the application of fungicides can reduce the production of volatile organic compounds in chickpea. Furthermore, volatile organic compounds (VOCs) can be employed for the evaluation of Ascochyta blight disease severity in chickpea crops (Kashyap and Kumar, 2021).

#### 2.2.2 VOCs in plant-plant interaction

Plants exhibiting resistance to herbivores and pathogens release volatile organic compounds (VOCs) that can prompt resistance reactions in nearby healthy plants. Latest investigations suggests that these VOCs may also induce resistance to pathogens through various mechanisms: either by priming the stimulated expression of resistance genes in the receiver plant or by inhibiting directly microbial pathogens, leading to a passive resistance in the plant subjected to VOCs (Sakurai et al., 2023). Research of Quintana-Rodriguez et al. (2015) revealed that VOCs released by infected resistant bean plants (Phaseolus vulgaris) offered resistance to bean anthracnose caused by Colletotrichum lindemuthianum in a susceptible cultivar after being exposed over 6 hours to volatile compounds collected from the headspace of resistant plants. Furthermore, individual VOCs like limonene, linalool, nonanal, methyl salicylate, and methyl jasmonate, at normal levels, could directly inhibit conidia development and can also suppress the conidia production using an active mycelium *in vitro*. VOCs from infected plants displayed a more potent inhibitory effect on conidial germination compared to VOCs from uninfected plants. This inhibitory effect was found to be correlated with the abundance of b-linalool, limonene, or methyl jasmonate. In conclusion, VOCs are pivotal in enhancing bean resistance to fungal pathogens, both by directly reinforcing the emitting plant’s resistance, and indirectly by influencing the resistance traits of nearby receiver plants through induced and associational resistance. Several VOCs like, linalool, limonene, nonanal, methyl salicylate, and methyl jasmonate, exhibit antimicrobial activity (Fernando et al., 2005; Arroyo et al., 2007; Neri et al., 2007). For instance, nonanal has been found to suppress fungal germination in leaves through direct fungistatic actions (Zeringue et al., 1996) and also conferred resistance in lima bean to Pseudomonas syringae (Yi et al., 2009). Notably, the dominant volatile component cis-hexenyl acetate in the volatile blend of the susceptible cultivar, is more probable to be linked to herbivory than pathogen resistance mechanism.

## 3 VOCs application on food legume crops

### 3.1 Exploring the role of VOCs in cropping systems

**Figure 6:**
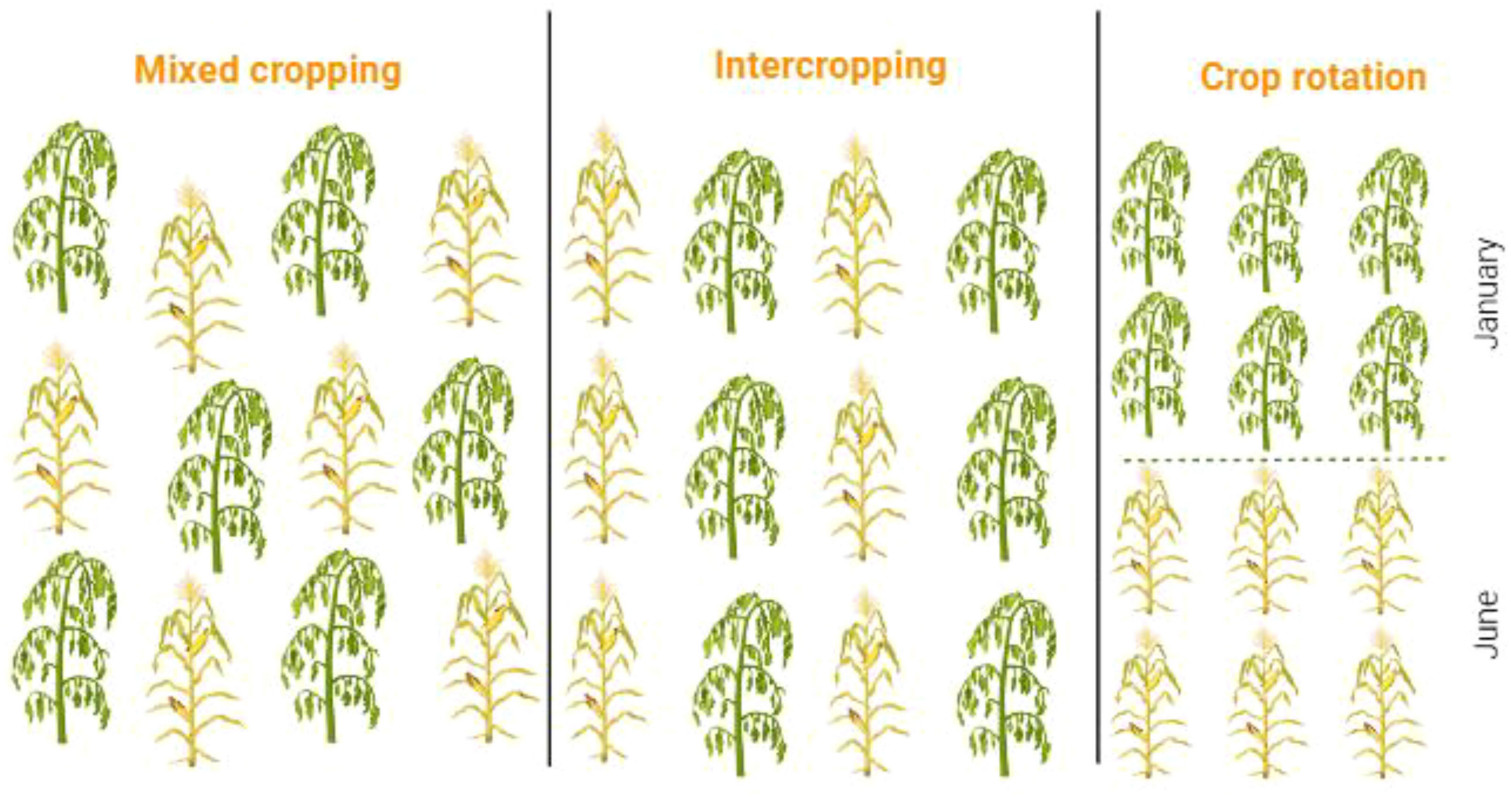
Different cropping systems adapted in legume crops as cultural practices to manage pest attack.

**Figure 7:**
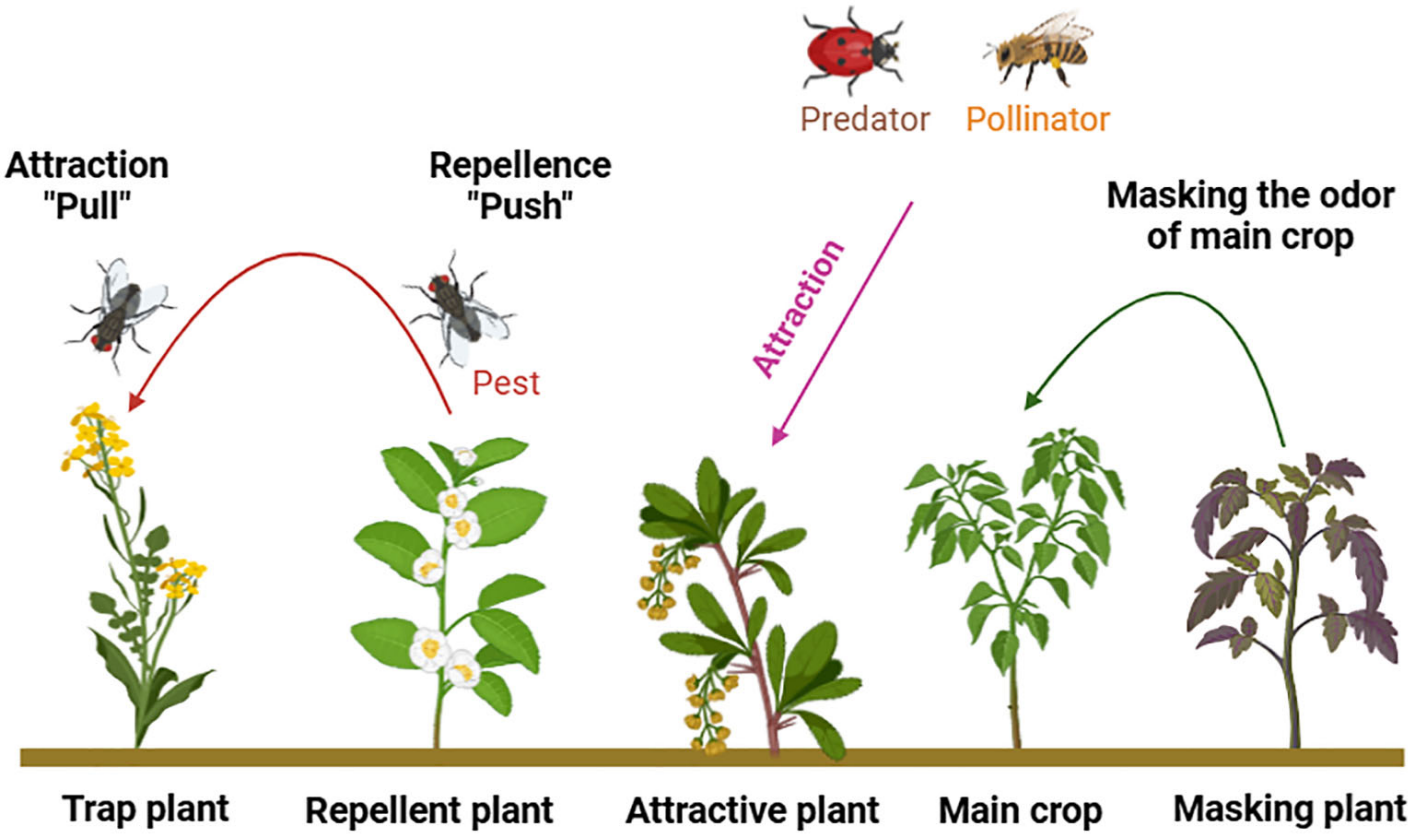
Various roles of service plants using different cropping systems for pest management and for other ecological services.

Smallholder farmers adopt diverse cropping systems to promote sustainable crop production (Brilli et al., 2019; Murphy-Bokern, 2022). Legume crops are integrated into crop rotation and multiple cropping strategies to reduce fertilizer consumption, minimize pesticide use, and enhance various ecosystem services (Agegnehu et al., 2008; Gaba et al., 2015; Wahbi et al., 2016; Ajal and Weih, 2022). Some major multiple cropping systems (**Figure 6**), focusing on volatile organic compounds (VOCs) role in pest management and other ecological services are briefly described (**Figure 7**).

#### 3.1.1 Crop rotation

Crop rotation is an agricultural strategy that involves the sequential planting of various crops in one parcel for a set period of time to enhance soil fertility and control pests and diseases.

The rotation of wheat with chickpea has proven beneficial in reducing Fusarium head blight, through volatile organic compounds (VOCs) released from legumes. Cruz et al. (2012) showed that VOCs produced from both wheat and rotation crops, particularly chickpea, appear to have a negative impact on pathogenic *Fusarium* which cause significant financial deficits in crop rotations based on wheat.

The findings further indicate that in chickpea-wheat rotation systems, *Fusarium* inoculum rates could be minimized by cultivating chickpea genotypes with elevated concentrations of 1-hexanol and trans-2-hexen-1-ol in both roots and shoots (Cruz et al., 2012).

#### 3.1.2 Polyculture

Farmers grow different crops in the same area to exploit resources and avoid risks due to pest outbreaks or weather variability. As opposed to monocropping, polyculture increases crop diversity that influences insect populations and diseases by reducing the population density of herbivores and promoting beneficial insects like pollinators and natural enemies, especially parasitoids. Growing diverse plant species in close proximity can contribute to insect control through various mechanisms, which differ according to the particular volatile proprieties of each plant (Shrivastava et al., 2010).

##### 3.1.2.1 Inter - and mixed cropping

These cropping practices are a type of polyculture in which crop varieties or different plant species are planted, in the same space at same time, as mixed or arranged in rows or strips (Ghaley et al., 2005; Pelzer et al., 2012). Cereal-legume inter/mixed cropping practices contribute positively to insect pests’ control (Mweke et al., 2020; Emery et al., 2021).

In maize-legume intercropping, the overall system dynamics can influence the presence of legume pests (Bukovinszky et al., 2004). For instance, intercropping bean plants with older and taller maize plants can significantly reduced black aphids (*Aphis fabae*) infestations (Ogenga-Latigo et al., 1992). Similarly, cowpea crops experienced a lower population of pod-sucking bugs when grown alongside with maize at particular percentages, in contrast to being cultivated alone (Olufemi et al., 2001). The intercropping of cereals and legumes has the potential to impact the insect diversity and population by changing foliage nitrogen concentration and modifying the plant taste for herbivores (Pierre et al., 2023).

##### 3.1.2.2 Companion crops

The cultivation of both non-commercial and main crops is a common practice that supports nutrient uptake, pollination, and various other benefits. Push-pull is the most popularized innovation in managing insect pests through companion cropping (Murage et al., 2015; Eigenbrode et al., 2016; Isgren et al., 2023).

Trap cropping is a sort of companion planting where specific plants are strategically grown around the field to protect the main crop by reducing pest pressure. Typically, trap crops exhibit higher attractiveness to insects compared to the main crops, and the allocated area for trap crops should be relatively small (Sarkar et al., 2018).

These companion plants (CP) are recognized to emit compounds that may impact the aphid behavior through various ways, including mobility, feeding, and reproductivity (Moreno and Racelis, 2015).

Firstly, volatile organic compounds (VOCs) released by CPs can entice aphids far from their primary host plants. Additionally, they have the ability to modify the perception of the host plant by masking its aroma which renders it undesirable host for aphids, or by emitting repellent volatiles. Thirdly, CP can attract natural enemies by releasing VOCs to enhance and maintain biological pest management (Ben-Issa et al., 2017).

In a research investigation on the black bean aphid (*A. fabae*) conducted by Nottingham et al., showed that combining host plant volatiles with α-pinene identified in rosemary oil (*Rosmarinus officinalis*) obscured the host plant odors and make them unattractive in olfactory tests, which disrupted the aphid’s behavioral response. Additionally, the individual volatile compound from CP, when tested alone, was repellent to the aphids.

The 3-butenyl or 4-pentenyl isothiocyanate released by savory (*Satureja hortensis*) and thyme (*Thymus vulgaris*) exhibited a similar obscuring impact on enticing host odors for the black bean aphid, but when tested independently, they proved to be repellent.

Repulsive plants, which are non-host plants nearby that disrupt the insect activity and deter feeding on host plants, have been identified in laboratory tests. The compounds myrtenal and isothiocyanates released from the *Brassicaceae* family, demonstrated repulsing effects on the black bean aphid (*A. fabae*). Moreover, many terpenoids present in CP-emitted VOCs (e.g., rosemary), like 1,8 cineole, α-pinene, or camphor were found to have a repellent impact on aphids.

Basedow et al. (2006) noticed that the intercropping arrangement of bean plants (*V. faba)* alongside basilic (*Ocimum basilicum)* and summer savory (*Satureja hortensis)* plants (*Lamiaceae* family) significantly reduced the *A. fabae* population in wind tunnels, greenhouses, and fields. Tests conducted by Nottingham et al. (1991), employing a straight-line pathway olfactometer and a flight cage to exhibit the deterrent impact of *Satureja hortensis* and *Tanacetum vulgare* on *A. fabae*. *Ocimum basilicum* also exhibited repellent activity against the black bean aphid.

### 3.2 The use of VOCs in crop breeding

In most cases, plant VOCs are genetically regulated and highly species specific (Splivallo et al., 2012; Niederbacher et al., 2015). Improved crop varieties, landraces and their wild relatives release various VOCs as a defense mechanism against biotic and abiotic stressors (Moayeri et al., 2007; Thomas et al., 2023). The variability in VOCs content can be an important trait to be exploited in developing germplasm for effective deployment in insect pest and disease management (Keneni et al., 2011; War et al., 2012; Araus and Cairns, 2014; Valencia-Ortiz et al., 2022).

Limited research showed that cultivated crops have less complex inducible volatiles due to modern breeding that make them less ecologically competitive comparing to their wild relatives (Palmgren et al., 2015; Rowen and Kaplan, 2016).

#### 3.2.1 VOCs and breeding for pest resistance

Differences in volatile organic compounds (VOCs) among genotypes offer insights into insect preferences and the degree of damage caused by insects. These findings help breeders to select genotypes that demonstrate higher tolerance or resistance to pest attacks, based on their VOCs profile. They can target crop germplasm that emits fewer herbivore-attractant volatile organic compounds (VOCs) or that increases the release of VOCs known to repel pests.

In cowpea, females of *C. maculatus* preferred some genotypes over the others in oviposition due to variation in volatile compounds. Ahuchaogu and Ojiako (2021) demonstrated how the mated female *C. maculatus* responded to odor signals released by multiple bean cultivars (Pinto beans, borno-brown beans and adzuki beans) employing both two-arm and four-arm olfactometers. The study of volatile organic compounds by gas chromatography coupled to mass spectrometry (GCMS) revealed variations in the abundance profile of these compounds. This implies that the nature and amounts of volatile compounds detected in beans impact the female *C. maculatus* behaviors related to host finding and preference. The volatile compounds like benzyl alcohol, nonanal, and limonene were identified as potential compounds capable to influence the behavioral attractiveness of beetles to specific bean types.

Moreover, the attractiveness of phytophagous alate *Aphis craccivora* (Hemiptera: Aphididae) in cowpea varies among cultivars of the same species depending on VOCs profile of each genotype. Diabate et al. (2019) identified 23 volatile compounds from four cowpea cultivars with just 4 volatiles (hexanal, 1‐octen‐3‐ol, (E)‐2‐hexenal, and p‐xylene) showing significant differences in emission quantities across the cultivars.

In the olfactometer assays, the aphids exhibited a notable preference for odors from cultivar Ex‐Luanda and significant non‐preference to Katumani 80. Machakos and Ken Kunde generated unbiased reactions.

The Ex‐Luanda cowpea cultivar appeared more attractive than Katumani 80 based on plant odor. The unattractant cultivar Katumani released significant amount of Hexanal and (E)‐2‐ hexenal, while the attractant cultivar Ex‐Luanda emitted large quantities of 1‐octen‐3‐ol and p‐xylene.

The composition and proportions of volatile organic compounds in cowpea varied among varieties. The attractant cowpea cultivar releases the adequate combination and particular proportion of compounds recognized by *A. craccivora* to identify the host plant, whereas the unattractive cultivar is distinguished via the unsuitable mixture and proportions of compounds.

In tests using standard compounds, *A. craccivora* responded neutrally once the attractant cowpea cultivar (Ex‐Luanda) was mixed with hexanal and (E)‐2‐hexenal compared to purify air. These two compounds may mask the volatile profile of Ex‐Luanda, making it less attractive.

In contrast, the appellant cultivar Ex‐Luanda was characterized by the presence of 1‐octen‐3‐ol compound. Although the existence of the two compounds 1‐octen‐3‐ol and p‐xylene with the less favored cultivar Katumani, *A. craccivora* didn’t display attraction.

In grass peas (*Lathyrus sativus* L.), Mitra et al. (2020) characterized the volatile organic compounds (VOCs) profile of two grass pea genotypes, namely BIO L 212 Ratan and Nirmal B-1, in response to infestation by the viviparous aphid *A. craccivora.*

The aphids attack led to an elevation in the VOCs emission in comparison to undamaged plants of each cultivar. Nevertheless, the overall quantity of VOCs was grater in the NIR cultivar in comparison with the BIO cultivar, for both undamaged or damaged plants. The overall quantity of VOCs released reflects the extent of insect damage (a greater number of aphids resulting in increased VOCs emission). The GC-MS analysis of the VOCs profile in damaged plants of the NIR cultivar revealed the presence of 4 compounds including thymol, benzyl alcohol, 1,3-diethylbenzene, and 1-hexadecene. In contrast, BIO grass peas cultivar plants exhibited 11 compounds such as benzyl alcohol, diacetone alcohol, p-cymene, linalool oxide, 1,3-diethylbenzene, acetophenone, 1-nonanol, ethylacetophenone, p-cymen-7-ol, thymol, and 1-hexadecene, in their volatile extracts. This blend of VOCs functioned as a lure for *A. craccivora*. In olfactometer bioassays, female aphids displayed a preference for an artificial mixture comprising 1,3-diethylbenzene, benzyl alcohol, 1-hexadecene, and thymol. This finding suggests the potential use of these compounds in developing lures for an effective control of this insect pest.

In chickpea, trichomes provide a potential resistance mechanism against the pod borer *Helicoverpa armigera* (Hübner), as highlighted by Brar and Signh (2017). Glandular trichomes, specialized hairs on plant surfaces, serve as physical barriers against herbivores and act as essential chemical defenses. These trichomes produce various secondary chemicals, including terpenoids, phenylpropenes, flavonoids, methyl ketones, acyl sugars, and defensive proteins. Terpenoids, a major component of volatile mixtures triggered by herbivore activity, serve a crucial function in attracting predators and parasitoids to plants infested by herbivore. Additionally, terpenoids contribute to direct defenses by acting as deterrents or repellents, with higher concentrations often proving toxic. For instance, the sesquiterpene (E)-β-farnesene released by glandular trichomes was found to drive away aphids (*Myzus persicae*), whereas their parasitoids, such as the hymenopteran *Diaeretiella rapae*, were drawn to (E)-β-farnesene.

Phenylpropenes play double functions, acting as defense mechanism against herbivores, and also serving as attractants for pollinators. Notably, eugenol and methylchavicol are mainly produced and retained in glandular trichomes. The use of synthesized eugenol resulted in morality and deterrence in Coleoptera species. Methyl ketones, a form of volatile compounds derived from fatty-acid, are particularly effective in defending plants against pests. Plants typically contain Methyl ketones with 7 to 15 carbons consist of 2-heptanone, 2-nonanone, 2-undecanone, 2-tridecanone and 2-pentadecanone (Glas et al., 2012; Xing et al., 2017; Kaur and Kariyat, 2020).

#### 3.2.2 VOCs and crop breeding for disease resistance

Evaluation of disease severity is a crucial phase in developing disease-resistant cultivars. Visual evaluation and classical plant phenotyping techniques is a common practice in plant breeding; nevertheless, these methods requires intensive training, destructive harvesting during precise phenological periods, and are both time consuming and expensive.

Due to their particular properties, plant VOCs are a promising tool that could be used as a rapid and non-destructive assessment of plant phenotypic traits (Jud et al., 2018).

Research carried out by Zhang et al. (2023) showed that sensing methods utilizing volatile organic compounds (VOCs) such as a field asymmetric ion mobility spectrometry (FAIMS) system could be an alternative solution for disease monitoring. This method was evaluated as a non-invasive and fast VOC-based phenotyping approach for the surveillance of Ascochyta blight disease intensity in chickpeas.

In addition, the high variability of VOC emissions between plant genotypes and species makes them great phenotypic markers to distinguish between different levels of disease resistance and resilience, which in consequence will contribute to enhance the effectiveness and productivity of crops (Niederbacher et al., 2015).

To evaluate resistance for Aphanomyces root rot disease, caused by *Aphanomyces euteiches* Drechs., which is a serious soil-borne disease affecting various crops, such as pea plant (*Pisum sativum* L.). Marzougui et al. (2022) investigated the reaction of two cultivars, Ariel (susceptible) and Hampton (elevated degrees of partial resistance). Disease progression was assessed non-intrusively at three distinct intervals (15, 20, and 30 days post-inoculation). VOCs emitted by both infested and healthy plants were gathered employing dynamic headspace sampling. Analysis via GC-FID and GC-MS revealed a profile of sixteen volatile compounds including 2-Propanone, 2-Butanone, 3-Pentanone, 3-Hexanone, Hexanal, (E)-2-Hexanal, (Z)-3-Hexen-1-ol, (E)-2-Hexen-1-ol, 1-Hexanol, 3-Heptanone, 2-Heptanone, 2-Octanone, (Z)-3-Hexenyl acetate, (E)-2-Hexenyl acetate, Nonanal, and Decanal. At 20 DPI, the Ariel cultivar exhibited a significant increase in hexanal emissions compared to the Hampton cultivar. Concurrently, the standardized proportional peak strength of the Ariel cultivar showed elevated emissions of (Z)-3-hexen-1-ol and (Z)-3-hexenyl acetate (Marzougui et al., 2022).

Several VOCs detected through non-destructive sampling presented significant correlations with visual disease ratings and relative chlorophyll level.

This study indicated that pea varieties with varied level of resistance and susceptibility to Aphanomyces root rot displayed differences in VOC blends across several time intervals and development phases. The observed differences in VOC emissions between cultivars underscore the potential of VOCs as biomarker-based phenotyping tools for distinguishing resistance levels in evaluated pea cultivars. Integrating VOC profiles with high-throughput VOC detection methods presents a promising unique approach for characterizing disease reactions in plants.

Plants exhibiting resistance to herbivores and pathogens release volatile organic compounds (VOCs) that can prompt resistance reactions in nearby healthy plants. Latest investigations suggests that these VOCs may also induce resistance to pathogens through various mechanisms: either by priming the stimulated expression of resistance genes in the receiver plant or by inhibiting directly microbial pathogens, leading to a passive resistance in the plant subjected to VOCs.

Research of Quintana-Rodriguez et al. (2015) revealed that VOCs released by infected resistant bean plants (*Phaseolus vulgaris)* offered resistance to bean anthracnose caused by *Colletotrichum lindemuthianum* in a susceptible cultivar after being exposed over 6 hours to volatile compounds collected from the headspace of resistant plants. Furthermore, individual VOCs like limonene, linalool, nonanal, methyl salicylate, and methyl jasmonate, at normal levels, could directly inhibit conidia development and can also suppress the conidia production using an active mycelium *in vitro*. VOCs from infected plants displayed a more potent inhibitory effect on conidial germination compared to VOCs from uninfected plants. This inhibitory effect was found to be correlated with the abundance of b-linalool, limonene, or methyl jasmonate (Quintana-Rodriguez et al., 2015).

In conclusion, VOCs are pivotal in enhancing bean resistance to fungal pathogens, both by directly reinforcing the emitting plant’s resistance, and indirectly by influencing the resistance traits of nearby receiver plants through induced and associational resistance. Several VOCs like, linalool, limonene, nonanal, methyl salicylate, and methyl jasmonate, exhibit antimicrobial activity (Fernando et al., 2005; Arroyo et al., 2007; Neri et al., 2007). For instance, nonanal has been found to suppress fungal germination in leaves through direct fungistatic actions (Zeringue et al., 1996) and also conferred resistance in lima bean to *Pseudomonas syringae* (Yi et al., 2009). Notably, the dominant volatile component cis-hexenyl acetate in the volatile blend of the susceptible cultivar, is more probable to be linked to herbivory than pathogen resistance mechanism.

## 4 Microbial VOCs in food legume crops

During plant-pathogen or plant-pest interactions, microbial antagonists can interrupt the developmental process of pest or pathogen. This may take place via parasitic activity, conflict for area and food, the secretion of hydrolytic enzymes (Punja and Utkhede, 2003), and the release of antimicrobial compounds, including volatiles (Vinale et al., 2006). These microbial VOCs emitted from microorganisms like bacteria and fungi (Korpi et al., 2009; Francis et al., 2010; Thorn and Greenman, 2012) have the ability to travel via both air and ground (Aochi and Farmer, 2005). Therefore, they present an ideal “infochemicals” that mediate interactions of microorganisms with their natural environment (Weisskopf et al., 2021; Morath et al., 2012), including regulation of symbiotic associations, phytotoxicity, enhancement of plant development, activation of plant defense responses against pathogen attack, and insect attractant or repellent activities (Morath et al., 2012; Lee et al., 2016). These characteristics could be utilized to create eco-friendly solutions such as biofertilizers and biopesticides to enhance plant productivity and protection (Kaddes et al., 2020; Soto et al., 2021).

Over a 100 bacteria and fungi emit microbial VOCs in the ground (Effmert et al., 2012), with around 250 fungal VOCs were reported to date (Duc et al., 2022).

Five classes of microbial volatiles (alcohols, ketones, aromatic compounds, terpenes, and organic acids) each comprised at least 10% of the volatiles, reaching 64% of the total diversity. Minor groups including aldehydes, alkanes, alkenes, furans, ester, sulfur and nitrogen combining substances, and ethers, contributing together to over 37% of the total diversity (Schenkel et al., 2015; Bennett and Inamdar, 2015).

Fungi produce various mixtures of volatile organic compounds (VOCs) that are originate from both primary and secondary metabolism processes (Korpi et al., 2009). The composition of these volatile blends can vary based on the fungi species, the intra- and interspecific interactions (Schulz-Bohm et al., 2015; Piechulla et al., 2017), and the growth conditions (temperature, pH, moisture level, substrate, nutrients, and duration of incubation) (Morath et al., 2012; Bennett et al., 2013; Plaszkó et al., 2020). For example, according to Savelieva et al. (2016), *Fusarium* fungi grown on potato sucrose agar medium (PSA) release a broader range and greater amounts of VOCs than those cultivated on autoclaved wheat kernels (WK). Schenkel et al. (2018) have also reported considerable variances in the VOC composition of the same fungal species when cultivated in soil or malt extract medium. Moreover, the work of Li et al. (2016) showed that VOC retention in soils is affected by various environmental parameters including, temperature, moisture content, and pH that determine the polarity of VOCs and alter their evaporation pressure.

Microbial VOCs can reduce disease severity by inhibiting mycelial growth, creating unfavorable conditions for the development of diseases, stimulating soil-borne biocontrol agents, activating defense responses, and priming plants against future pathogen or pest attacks (Köhl et al., 2019).

One of the initial instances illustrating the inhibitory impact of microbial VOCs on plant pathogens were those generated by *Pseudomonas* isolates obtained from soybean and canola, which engaged in restraining and minimizing mycelial development of *Sclerotinia sclerotiorum* (Fernando et al., 2005).

Besides, VOCs released by two endophytic *Bacillus* have notably decreased both the mass and quantity of *S. sclerotiorum* persistent forms (sclerotia) (Massawe et al., 2018; Li et al., 2019). Similarly, research conducted by Fialho et al. (2011) demonstrated that the VOCs mixture emitted by *S. cerevisiae*, comprises alcohols like 3-methyl-1-butanol, ethanol, 2-methyl-1-butanol, and phenylethyl alcohol, and esters such as ethyl acetate and ethyl octanoate, have an effective control over *S. sclerotiorum* both *in vitro* and on bean seeds.

The substances 2-methyl-1-butanol, and 3-methyl-1-butanol showed the highest efficacy towards *S. sclerotiorum*, completely reducing its mycelial development at a concentration of 0.8 µL mL^-1^, followed by ethyl acetate, at 1.2 µL mL^-1^. Fumigating bean seeds with *S. cerevisiae* VOCs at 3.5 µL mL^-1^ resulted in 75% decrease in *S. sclerotiorum* occurrence post 4 days.

*Trichoderma* species have long been considered one of the most promising biocontrol agents; they are capable to generate a wide range of secondary metabolites that could influence the process of their biological function (Li et al., 2016). The research work of Kumar et al. (2019) showed that VOCs emitted by three *Trichoderma* species *Trichoderma harzianum*, *Trichoderma viride*, and *Trichoderma konigii* have hindered the mycelium development of *Fusarium oxysporum* f. sp. *ciceri* by 79.25%, 62.27%, and 50%, respectively. Also, species of *Streptomyces* have exhibited great potential for controlling plant fungal diseases by producing antifungal compounds (Li et al., 2012). Research carried out by Amini et al. (2016) showed that the antagonistic bacteria *Streptomyces* spp. has strong inhibitory effects against chickpea wilt. The isolated strains of Streptomyces (KS55, KS58, KS112, KS62, and KS31) produced a mixture of VOCs that can reduce disease severity by inhibiting mycelial growth of the pathogen, ranging from 20.2 to 33.4%.

Moreover, Elbouazaoui et al. (2022) revealed that volatile compounds produced by *Bacillus* can inhibit the development of *Fusarium oxysporum* f. sp. *ciceri*, with a high percentage of inhibition exceeding 30% obtained from *B. subtilis*.

Concerning the role of VOCs in inducing plant defenses, Ballhorn et al. (2013) investigated the impact of rhizobial symbiosis on volatile production in lima bean plants (Fabaceae: *Phaseolus lunatus* L.) and their role in legume defenses against herbivores.

They have demonstrated that the volatile organic compounds stimulated by jasmonic acid in rhizobia-colonized lima bean plants have repellent effects on the specialist herbivore pest (Mexican bean beetle; *Coccinellidae*: *Epilachna varivestis*). Results revealed that rhizobial symbiosis can induce plant defense via volatile production and can affect the choice behavior of beetles, so it can serve as a fundamental component of legume defenses against herbivores.

Additionally, VOCs produced by Lima bean plants colonized by rhizobia, a nitrogen-fixing bacteria, showed a repellent action on the Mexican bean beetles (Epilachna varivestis), which resulted in less damage from this pest (Bustos-Segura et al., 2024).

Microbial VOC can also control harmful plants and weed. For example, volatile compounds produced by fungi including 3-methyl-1-butanol, 1-octen-3-ol, 2-phenylethanol, 3-octanol, 1-hexanol, 3- octanone, and trans-2-octenal are categorized as toxic against plants. These compounds produced by different fungi can affect negatively the root growth and seed germination (Duc et al., 2022).

## 5 Conclusion and futures perspectives

In conclusion, the exploration of volatile organic compounds (VOCs) in the management of insect pests and diseases of food legumes holds significant promise and potential.

This assessment underscores the promising role of VOCs in enhancing disease and pest management strategies for cool season food legumes, offering a more sustainable and eco-friendly alternative to traditional control methods. Our research highlights several key findings and advancements in this field. The dynamic role of VOCs in mediating plant responses to biotic stresses, enabling plant to inhibit disease development, induce resistance to pathogens or herbivores, and control pest population in the field by acting as traps. Additionally, the attractive and repellent properties of plant VOCs are exploited in cropping systems of various food legumes, like bean plants, to either repel pest or attract natural enemies which reduce reliance on chemical pesticides and minimize production costs. Furthermore, traditional breeding programs that focus on genetic traits for resistance in food legumes can benefit from using VOCs as biomarkers for rapid, non-invasive plant phenotyping VOCs can also serve as diagnostic tool for an early disease or pest detection, which would allow grower to proactively intervene and select the appropriate control strategy ultimately, reducing yield losses and pesticides use. Integrating plant and microbial VOCs into crop management strategies represents a significant step and a cost-effective solution forward in sustainable agriculture, potentially improving yield quality and crop productivity.

Despite the encouraging prospects, challenges such as environmental sensitivity, inherent reactivity, and low concentrations of VOCs pose obstacles to their widespread implementation. Overcoming these challenges requires continued research to develop advanced analysis techniques, understand the factors influencing VOC production, and their mechanisms of action.

In summary, the use of VOCs in pest and disease management represents an exciting and promising area of research. While there are still many challenges to overcome, continued research has the potential to lead to the development of new, sustainable, and eco-friendly methods for protecting crops and increasing yield.

## References

[B1] Adane A. Vetten H. J. (2022). Chickpea chlorotic stunt virus : a threat to cool-season food legumes. Arch. Virol. 167 (1). doi: 10.1007/s00705-021-05288-4 34729666

[B2] Adhikary P. Mukherjee A. Barik A. (2015). Attraction of callosobruchus maculatus (F.) (Coleoptera: Bruchidae) to four varieties of lathyrus sativus l. seed volatiles. Bull. Entomol. Res. 105 (2), 187–201. doi: 10.1017/S000748531400087X 25524148

[B3] Agegnehu G. Ghizaw A. Sinebo W. (2008). Yield potential and land-use efficiency of wheat and faba bean mixed intercropping. Agron. Sustain. Dev. 28, 257–263. doi: 10.1051/agro:2008012

[B4] Ahuchaogu C. E. Ojiako F. O. (2021). Host seed type and volatile compound abundance level mould host location and preference behaviours in *Callosobruchus maculatus* (Fabricius 1775) (Coleoptera: Chrysomelidae). Polish J. Entomol. 90, 152–163. doi: 10.5604/01.3001.0015.4380

[B5] Ait Taadaouit N. El Fakhouri K. Sabraoui A. Maalouf F. Rohi L. El Bouhssini M. (2021a). First sources of resistance in faba bean (*Vicia faba* L.) to the stem borer weevil, *Lixus algirus* L. (Coleoptera: Curculionidae). Phytoparasitica. 49, 349–356. doi: 10.1007/s12600-021-00885-0

[B6] Ait Taadaouit N. El Fakhouri K. Sabraoui A. Rohi L. El Bouhssini M. (2021b). *Lixus algirus* L. (Coleoptera: Curculionidae): biology, population fluctuation, infestation as affected by varieties, location, and planting dates in Morocco. J. Entomol. Acarol. Res. 53, 9324. doi: 10.4081/jear.2021.9324

[B7] Ajal J. Weih M. (2022). Nutrient accumulation pattern in mixtures of wheat and faba bean is strongly influenced by cultivar choice and co-existing weeds. Biology 11, 630. doi: 10.3390/2Fbiology11050630 35625358 PMC9137686

[B8] Ajayi O. E. Balusu R. Morawo T. O. Zebelo S. Fadamiro H. (2015). Semiochemical modulation of host preference of *Callosobruchus maculatus* on legume seeds. J. Stored Prod. Res. 63, 31–37. doi: 10.1016/j.jspr.2015.05.003

[B9] Amini J. Agapoor Z. Ashengroph M. (2016). Evaluation of Streptomyces spp. against Fusarium oxysporum f. sp. *ciceris* for the management of chickpea wilt. J. Plant Prot. Res. 56, (3). doi: 10.1515/jppr-2016-0038

[B10] Annaz H. El Fakhouri K. Ben Bakrim W. Mahdi I. El Bouhssini M. Sobeh M. (2023). Bergamotenes: a comprehensive compile of their natural occurrence, biosynthesis, toxicity, therapeutic merits and agricultural applications. Crit. Rev. Food Sci. Nutr. doi: 10.1080/10408398.2023.2184766 36876517

[B11] Aochi Y. O. Farmer W. J. (2005). Impact of soil microstructure on the molecular transport dynamics of 1,2-dichloroethane. Geoderma. 127, 137–153. doi: 10.1016/j.geoderma.2004.11.024

[B12] Araus J. L. Cairns J. E. (2014). Field high-throughput phenotyping: the new crop breeding frontier. Trends Plant Sci. 19, 52–61. doi: 10.1016/j.tplants.2013.09.008 24139902

[B13] Arroyo F. T. Moreno J. Daza P. Boianova L. Romero F. (2007). Antifungal activity of strawberry fruit volatile compounds against *Colletotrichum acutatum* . J. Agric. Food Chem. 55, 5701–5707. doi: 10.1021/jf0703957 17567029

[B14] Aznar-Fernández T. Rubiales D. (2019). Flower and pod source influence on pea weevil (*Bruchus pisorum*) oviposition capacity and preference. Front. Plant Sci. 10. doi: 10.3389/fpls.2019.00491 PMC6491779 31068956

[B15] Baldwin I. T. (2010). Plant volatiles. Curr. Biol. 20, R392–R397. doi: 10.1016/j.cub.2010.02.052 20462477

[B16] Ballhorn D. J. Kautz S. Schädler M. (2013). Induced plant defense via volatile production is dependent on rhizobial symbiosis. Oecologia. 172, 833–846. doi: 10.1007/s00442-012-2539-x 23242424

[B17] Barney J. N. Sparks J. P. Greenberg J. Whitlow T. H. Guenther A. (2009). Biogenic volatile organic compounds from an invasive species: impacts on plant–plant interactions. Plant Ecol. 203, 195–205. doi: 10.1007/s11258-008-9529-4

[B18] Basedow T. Hua L. Aggarwal N. (2006). The infestation of vicia faba l. (Fabaceae) by aphis fabae (Scop.) (Homoptera: Aphididae) under the influence of lamiaceae (Ocimum basilicum l. and satureja hortensis l.). J. Pest Sci. 79, 149–154. doi: 10.1007/s10340-006-0128-7

[B19] Ben-Issa R. Gomez L. Gautier H. (2017). Companion plants for aphid pest management. Insects 8 (4), 112. doi: 10.3390/insects8040112 29053585 PMC5746795

[B20] Bennett J. W. Hung R. Lee S. Padhi S. (2013). Fungal and bacterial volatile organic compounds; an overview and their role as ecological signaling agents. The Mycota IX Fungal Interactions (Berlin/Heidelberg, Germany: The Mycota IX Fungal Interactions), 373–393. doi: 10.1007/978-3-642-30826-0_18

[B21] Bennett J. W. Inamdar A. A. (2015). Are some fungal volatile organic compounds (VOCs) mycotoxins? Toxins (Basel). 7 (9), 3785–3804. doi: 10.3390/toxins7093785 26402705 PMC4591661

[B22] Bezerra R. Souto L. Sant'Ana A. Ambrogi B. (2021). Indirect plant defenses: volatile organic compounds and extrafloral nectar. Arthropod-Plant Interact. 15 (4). doi: 10.1007/s11829-021-09837-1

[B23] Boulamtat R. Lhaloui S. Sabraoui A. El-Fakhouri K. Oubayoucef A. Mesfioui A.. (2019). Antifeedant and larvicidal activities of *Mentha pulegium* on chickpea pod borer *Helicoverpa armigera* (Lepidoptera: Noctuidae). Int. J. Trop. Insect Sci. 40, 151–156. doi: 10.1007/s42690-019-00064-z

[B24] Brar H. S. Singh R. (2017). Role of trichomes on leaves and pods for imparting resistance in chickpea [Cicer arientinum (L.)] genotypes against helicoverpa armigera. J. Appl. Nat. Sci. 9, 2193–2198. doi: 10.31018/JANS.V9I4.1509

[B25] Brilli F. Loreto F. Baccelli I. (2019). Exploiting plant volatile organic compounds (VOCs) in agriculture to improve sustainable defense strategies and productivity of crops. Front. Plant Sci. 10. doi: 10.3389/fpls.2019.00264 PMC6434774 30941152

[B26] Bruce T. J. A. Martin J. L. Smart L. E. Pickett J. A. (2011). Development of semiochemical attractants for monitoring bean seed beetle, *Bruchus rufimanus* . Pest Manage. Science. 67, 1303–1308. doi: 10.1002/ps.2186 21538800

[B27] Bruno D. Grossi G. Salvia R. Scala A. Farina D. Grimaldi A.. (2018). Sensilla morphology and complex expression pattern of odorant binding proteins in the vetch aphid *megoura viciae* (Hemiptera: aphididae). Front. Physiol. 9, 777. doi: 10.3389/fphys.2018.00777 29988577 PMC6027062

[B28] Bukovinszky T. Tréfás H. van Lenteren J. C. Vet L. E. M. Fremont J. (2004). Plant competition in pest-suppressive intercropping systems complicates evaluation of herbivore responses. Agric. Ecosyst. Environ. 102 (2), 185–196. doi: 10.1016/j.agee.2003.08.008

[B29] Bustos-Segura C. Godschalx A. L. Malacari L. Deiss F. Rasmann S. Ballhorn D. J.. (2024). Rhizobia-legume symbiosis mediates direct and indirect interactions between plants, herbivores and their parasitoids. Heliyon. 10 (6), e27815. doi: 10.1016/j.heliyon.2024.e27815 38524601 PMC10957422

[B30] Calles T. (2016). The International Year of Pulses: what are they and why are they important. Agric. Dev. 26, 40–42.

[B31] Carrillo-Perdomo E. Raffiot B. Ollivier D. (2018). Identification of novel sources of resistance to seed weevils (*Bruchus* spp.) in a faba bean germplasm collection. Front. Plant Sci. 10. doi: 10.3389/fpls.2018.01914 PMC6333698 30687341

[B32] Castro A. M. Tapias J. Ortiz A. Benavides P. Góngora C. E. (2017). Identification of attractant and repellent plants to coffee berry borer, *Hypothenemus hampei* . Entomologia Experimentalis Applicata 164, 120–130. doi: 10.1111/eea.2017.164.issue-2

[B33] Ceballos R. Fernández N. Zúñiga S. Zapata N. (2015). Electrophysiological and behavioral responses of pea weevil *Bruchus pisorum* L. (Coleóptera: Bruchidae) to volatiles collected from its host *Pisum sativum* L. Chilean J. Agric. Res. 75, 202–209. doi: 10.4067/S0718-58392015000200009

[B34] Choudhary D. K. Johri B. N. Prakash A. (2008). Volatiles as priming agents that initiate plant growth and defence responses. Curr. Sci. 94, 595–604.

[B35] Chrigui N. Sari D. Sari H. (2020). Introgression of Resistance to Leaf miner (*Liriomyza cicerina* Rondani) from *Cicer reticulatum* Ladiz. to *C. arietinum* L. and Relationships between Potential Biochemical Selection Criteria. Agronomy. 11, 57. doi: 10.3390/agronomy11010057

[B36] Congdon B. S. Coutts B. A. Renton M. Flematti G. R. Jones R. A. C. (2017). Establishing alighting preferences and species transmission differences for Pea seed-borne mosaic virus aphid vectors. Virus Res. 241, 145–155. Available at: https://www.sciencedirect.com/science/article/pii/S0168170217301764. 28408208 10.1016/j.virusres.2017.04.005

[B37] Cruz A. F. Hamel C. Yang C. (2012). Phytochemicals to suppress Fusarium head blight in wheat–chickpea rotation. Phytochemistry. 78, 72–80. doi: 10.1016/j.phytochem.2012.03.003 22520499

[B38] Dell'Aglio D. D. Tayeh N. (2023). Responsiveness of the broad bean weevil, Bruchus rufimanus, to Vicia faba genotypes. Entomologia Experimentalis Applicata. 171, 312–322. doi: 10.1111/eea.13277

[B39] Delory B. M. Delaplace P. Fauconnier M. L. Du Jardin P. (2016). Root-emitted volatile organic compounds: can they mediate belowground plant-plant interactions? Plant Soil 402, 126. doi: 10.1007/s11104-016-2823-3

[B40] Devrnja N. Milutinovic M. Savic J. (2022). When scent becomes a weapon— plant essential oils as potent bioinsecticides. Sustainability 14, 6847. doi: 10.3390/su14116847

[B41] Diabate S. Deletre E. Murungi L. K. (2019). Behavioural response of alate *Aphis craccivora* Koch (Homoptera: Aphididae) to volatiles from different cowpea cultivars. J. Appl. Entomol. 143, 659–669. doi: 10.1111/jen.12633

[B42] Dicke M. Loreto F. (2010). Induced plant volatiles: from genes to climate change. Trends Plant Sci. 15, 115–117. doi: 10.1016/j.tplants.2010.01.007 20137997

[B43] Duc N. G. Ha T. N. V. van Doan C. Hamow H. A. (2022). Volatile organic compounds shape belowground plant–fungi interactions. Front. Plant Sci. 13. doi: 10.3389/fpls.2022.1046685 PMC9763900 36561453

[B44] Dudareva N. Negre F. Aharoni A.. (2004). Plant volatiles: Recent advances and future perspectives. Crit. Rev. Plant Sci. 23 (5), 539–558.

[B45] Effmert U. Kalderás J. Warnke R. Piechulla B. (2012). Volatile mediated interactions between bacteria and fungi in the soil. J. Chem. Ecol. 38 (6), 665–703. doi: 10.1007/s10886-012-0135-5 22653567

[B46] Eigenbrode S. D. Birch A. N. E. Lindzey S. Meadow R. Snyder W. E. Pocock M. (2016). Review: A mechanistic framework to improve understanding and applications of pushpull systems in pest management. J. Appl. Ecol. 53, 202–212. doi: 10.1111/jpe.2016.53.issue-1

[B47] Elbouazaoui A. Douira A. Maafa I. Ahmed S. K. (2022). Integrating sowing date with chickpea genotypes in managing fusarium wilt in Morocco. Agriculture. 12, 773. doi: 10.3390/agriculture12060773

[B48] El Fakhouri K. Boulamtat R. Sabraoui A. El Bouhssini M. (2022). The chickpea pod borer, *helicoverpa armigera* (Hübner): yield loss estimation and biorational insecticide assessment in Morocco. Agronomy. 12, 3017. doi: 10.3390/agronomy1212301

[B49] El Fakhouri K. Huang J. Sabraoui A. Aasfar A. El Bouhssini M. Gut L. (2021). Screening of volatile compounds used in host location by the faba bean stem borer, Lixus algirus on faba bean in Morocco.

[B50] Emery S. E. Anderson P. Carlsson G. (2021). The potential of intercropping for multifunctional crop protection in oilseed rape (*Brassica napus* L.). Front. Agron. 3. doi: 10.3389/fagro.2021.782686

[B51] Fernando W. G. D. Ramarathan R. Krishnamoorthy A. S. Savchuk S. C. (2005). Identification and use of potential bacteria organic antifungal volatile isolates in biocontrol. Soil Biol. Biochem. 37, 955–964. doi: 10.1016/j.soilbio.2004.10.021

[B52] Fialho M. B. Heloisa M. Moraes D. Tremocoldi A. R. Pascholati S. F. (2011). Potential of antimicrobial volatile organic compounds to control *Sclerotinia sclerotiorum* in bean seeds. Pesq. Agropec. Bras. Brasília. 46, 137–142. doi: 10.1590/S0100-204X2011000200004

[B53] Ficke F. Asalf B. Norli H. R. (2021). Volatile organic compound profiles from wheat diseases are pathogen-specific and can be exploited for disease classification. Front. Microbiol. 12. doi: 10.3389/fmicb.2021.803352 PMC8776713 35069508

[B54] Fountain M. T. Baroffio C. Borg-Karlson A. K. Brain P. Cross J. V. Farman D. I.. (2017). Design and deployment of semiochemical traps for capturing *Anthonomus rubi* Herbst (Coleoptera: Curculionidae) and *Lygus rugulipennis Poppius* (Hetereoptera: Miridae) in soft fruit crops. Crop Prot. 99, 1–9. doi: 10.1016/j.cropro.2017.05.001

[B55] Francis I. Holsters M. Vereecke D. (2010). The gram-positive side of plant-microbe interactions. Environ. Microbiol. 12, 1–12. doi: 10.1111/j.1462-2920.2009.01989.x 19624707

[B56] Gaba S. Lescourret F. Boudsocq S. (2015). Multiple cropping systems as drivers for providing multiple ecosystem services from concepts to design. Agron. Sustain. Dev. 35, 607–623. doi: 10.1007/s13593-014-0272-z

[B57] Gaur P. M. Jukanti A. K. Varshney R. (2012). Impact of genomic technologies on chickpea breeding strategies. Agron. J. 2, 199–221. doi: 10.3390/agronomy2030199

[B58] Ge J. Li N. Yang J. (2019). Female adult puncture-induced plant volatiles promote mating success of the pea leaf miner via enhancing vibrational signals. Phil. Trans. R. Soc. B374, 20180318. doi: 10.1098/rstb.2018.0318 PMC6367149 30967018

[B59] Ghaley B. B. Hauggaard-Nielsen H. Høgh-Jensen H. (2005). Intercropping of wheat and pea as influenced by nitrogen fertilization. Nutr. Cycl Agroecosyst 73, 201–212. doi: 10.1007/s10705-005-2475-9

[B60] Glas J. J. Schimmel B. C. J. Alba J. M. (2012). Plant glandular trichomes as targets for breeding or engineering of resistance to herbivores. Int. J. Mol. Sci. 13, 17077–17103. doi: 10.3390/ijms131217077 23235331 PMC3546740

[B61] Gualtieri L. Monti M. M. Mele F. Russo A. Pedata P. A. Ruocco M. (2022). Volatile organic compound (VOC) profiles of different trichoderma species and their potential application. J. Fungi. 8, 989. doi: 10.3390/jof8100989 PMC9605199 36294554

[B62] Guerrieri E. Digilio M. C. (2008). Aphid-plant interactions: a review. J. Plant Interactions. 3, 223–232. doi: 10.1080/17429140802567173

[B63] Hamdi S. H. Abidi S. Sfayhi D. (2017). Nutritional alterations and damages to stored chickpea in relation with the pest status of *Callosobruchus maculatus* (Chrysomelidae). J. Asia-Pacific Entomol. 20, 1067–1076. doi: 10.1016/j.aspen.2017.08.008

[B64] Haware M. P. Nene Y. L. (1982). Symptomless carriers of the chickpea wilt Fusarium. Plant Dis. 66, 809–810. doi: 10.1094/PD-66-809

[B65] Hegde M. Oliveira J. N. da Costa J. G. Bleicher E. Santana A. E. G. Bruce T. J. A.. (2011). Identification of Semiochemicals Released by Cotton, Gossypium hirsutum, Upon Infestation by the Cotton Aphid, Aphis gossypii. J. Chem. Ecol. 37, 741–750. doi: 10.1007/s10886-011-9980-x 21671083

[B66] Horiuchi J. I. Badri D. V. Kimball B. A. Negre F. Dudareva N. Paschke M. W.. (2007). The floral volatile, methyl benzoate, from snapdragon (*Antirrhinum majus*) triggers phytotoxic effects in Arabidopsis thaliana. Planta. 226, 1–10. doi: 10.1007/s00425-006-0464-0 17216481

[B67] Houasli C. Sahri A. Nsarellah N. (2021). Chickpea (*Cicer arietinum* L.) breeding in Morocco: genetic gain and stability of grain yield and seed size under winter planting conditions. Euphytica. 217, 159. doi: 10.1007/s10681-021-02885-x

[B68] InsectImages (2018). Insect images : The source for entomology photos (The University of Georgia).

[B69] Isgren E. Clough Y. Murage A. (2023). Are agricultural extension systems ready to scale up ecological intensification in East Africa? A literature review with particular attention to the Push−Pull Technology (PPT). Food Security. 15, 1399–1420. doi: 10.1007/s12571-023-01387-z

[B70] Jones R. A. C. Roger A. C. (2021). Global plant virus disease pandemics and epidemics. Plants. 10, 233. doi: 10.3390/plants10020233 33504044 PMC7911862

[B71] Jud W. Winkler J. B. Niederbacher B. (2018). Volatilomics: a non-invasive technique for screening plant phenotypic traits. Plant Methods 14, 109. doi: 10.1186/s13007-018-0378-4 30568721 PMC6297985

[B72] Kaddes A. Fauconnier M. L. Sassi K. Berhal C. Nasraoui B. Jijakli H. (2020). Efficacité des Composés Organiques Volatils fongiques (synthèse bibliographique). Biotechnol. Agron. Société Environ. 24, 81–98. doi: 10.25518/1780-4507.18531

[B73] Karolkowski A. Guichard E. Briand L. (2021). Volatile compounds in pulses: A review. Foods 10, 3140. doi: 10.3390/foods10123140 34945691 PMC8702198

[B74] Kashyap B. Kumar R. (2021). Sensing methodologies in agriculture for monitoring biotic stress in plants due to pathogens and pests. Inventions 6, 29. doi: 10.3390/inventions6020029

[B75] Kasote D. Lee J. Sreenivasulu N. (2023). Editorial: Volatilomics in plant and agricultural research: recent trends. Front. Plant Sci. 14, 1289998. doi: 10.3389/fpls.2023.1289998 37841633 PMC10570788

[B76] Kaur J. Kariyat R. (2020). “Role of Trichomes in Plant Stress Biology,” in Evolutionary Ecology of Plant-Herbivore Interaction. Eds. Núñez-Farfán J. Valverde P.. (Cham: Springer), 15–35. doi: 10.1007/978-3-030-46012-9_2

[B77] Keneni K. Bekele B. Getu E. Imtiaz M. Damte T. Mulatu M.. (2011). Breeding food legumes for resistance to storage insect pests: potential and limitations. Sustainability 3, 1399–1415. doi: 10.3390/su3091399

[B78] Köhl J. Kolnaar R. Ravensberg W. J. (2019). Mode of action of microbial biological control agents against plant diseases: relevance beyond efficacy. Front. Plant Sci. 10. doi: 10.3389/fpls.2019.00845 PMC6658832 31379891

[B79] Kong C.-H. Li Z. Li F.-L. Xia X.-X. Wang P. (2024). Chemically mediated plant–plant interactions: Allelopathy and allelobiosis. Plants 13, 626. doi: 10.3390/plants13050626 38475470 PMC10934397

[B80] Korpi A. Jarnberg J. Pasanen A. L. (2009). Microbial volatile organic compounds. Crit. Rev. Toxicol. 39, 139–193. doi: 10.1080/10408440802291497 19204852

[B81] Kramer R. Abraham W. R. (2012). Volatile sesquiterpenes from fungi: What are they good for? Phytochem 11, 15–37. doi: 10.1007/s11101-011-9216-2

[B82] Krimi S. B. Ahmed S. Imtiaz M. Hamwieh A. Udupa S. M. Sahri A.. (2022). Pathogen diversity and mating types of *Didymella rabiei* isolates collected from Morocco. Curr. Plant Biol. 29, 100231. doi: 10.1016/j.cpb.2021.100231

[B83] Kumar M. Kumar V. Rana M. Srivastava S. (2019). Effect of volatile and nonvolatile compounds of Trichoderma spp. Against Fusarium isolates causing chickpea wilt in Punja. Plant Arch. 19, 159–162.

[B84] Lee S. Yap M. Behringer G. Hung R. Bennett J. W. (2016). Volatile Organic Compounds emitted by Trichoderma species mediate plant growth. Fungal Biol. Biotechnol. 3, 7. doi: 10.1186/s40694-016-0025-7 28955466 PMC5611631

[B85] Li F. Tang M. Tang X. Sun W. Gong J. Yi. Y. (2019). *Bacillus subtilis*-*Arabidopsis thaliana*: a model interaction system for studying the role of volatile organic compounds in the interchange between plants and bacteria. Botany 97, (12). doi: 10.1139/cjb-2019-0093

[B86] Li Q. Ning P. Zheng L. Huang J. Li G. Hsiang T. (2012). Effects of volatile substances of Streptomyces globisporus JK-1 on control of Botrytis cinerea on tomato fruit. Biol. Control 61, 113–120. doi: 10.1016/j.biocontrol.2011.10.014

[B87] Li G. Su H. Li X. Kuhn U. Meusel H. Hoffmann T.. (2016). Uptake of gaseous formaldehyde by soil surfaces: a combination of adsorption/desorption equilibrium and chemical reactions. Atmos. Chem. Phys. 16, 10299–10311. doi: 10.5194/acp-16-10299-2016

[B88] Lokesh B. K. Shashidhara N. Kantharaju V. (2020). Survey for the incidence of wilt disease and management of wilt in chickpea. Int. J. Agric. Sci. 16, 91–94. doi: 10.15740/HAS/IJAS/16.1/91-94

[B89] Maffei M. E. Mithofer A. Boland W. (2007). Insects feeding on plants: rapid signals and responses preceding the induction of phytochemical release. Phytochemistry. 68, 22-24, 2946–2959. doi: 10.1016/j.phytochem.2007.07.016 17825328

[B90] Makhlouf L. El Fakhouri K. Kemal S. A. Maafa I. Meftah Kadmiri I. El Bouhssini M. (2024). Advances in analytical techniques for assessing volatile organic compounds in pulse crops: a comprehensive review. Front. Hortic. 3, 1394041. doi: 10.3389/fhort.2024.1394041 PMC11486643 39430890

[B91] Makkouk K. M. (2020). Plant pathogens which threaten food security: viruses of chickpea and other cool season legumes in West Asia and North Africa. Food Sec. 12, 495–502. doi: 10.1007/s12571-020-01017-y

[B92] Mario M. B. Astuti L. P. Jue-Liang H. (2023). Bioefficacy of eight different plant powders applied as fumigants against the adzuki bean weevil, *Callosobruchus chinensis* . Crop Prot. 167, 6200. doi: 10.1016/j.cropro.2023.106200

[B93] Marzougui A. Rajendran A. Mattinson D. S. Ma Y. McGee R. Garcia-Perez M.. (2022). Evaluation of biogenic markers-based phenotyping for resistance to Aphanomyces root rot in field pea. Inf. Process. Agric. 9 (1), 1–10. doi: 10.1016/j.inpa.2021.01.007

[B94] Massawe V. Hanif A. Farzand A. Mburu D. K. Ochola S. Wu L.. (2018). Volatile organic compounds of endophytic Bacillus spp. have biocontrol activity against sclerotinia sclerotiorum. Phytopathology. 108 (12). doi: 10.1094/PHYTO-04-18-0118-R 29927356

[B95] Mendesil E. Rämert B. Marttila S. (2016). Oviposition Preference of Pea Weevil, Bruchus pisorum L. Among Host and Non-host Plants and its Implication for Pest Management. Front. Plant Sci. 6. doi: 10.3389/fpls.2015.01186 PMC4702147 26779220

[B96] Mitra P. Das S. Debnath R. (2020). Identification of *Lathyrus sativus* plant volatiles causing behavioral preference of *Aphis craccivora* . Pest Manage. Sci. 77, 285–299. doi: 10.1002/ps.6018 32696596

[B97] Moayeri H. R. S. Ashouri A. Poll L. Enkegaard A. (2007). Olfactory response of a predatory mirid to herbivore induced plant volatiles: multiple herbivories vs. single herbivory. J. Appl. Entomol. 131, 326–332. doi: 10.1111/j.1439-0418.2007.01177.x

[B98] Morath S. U. Hung R. Bennett J. W. (2012). Fungal volatile organic compounds: A review with emphasis on their biotechnological potential. Fungal Biol. Rev. 26, 73–83. doi: 10.1016/j.fbr.2012.07.001

[B99] Moreno C. R. Racelis A. E. (2015). Attraction, repellence, and predation: role of companion plants in regulating *myzus persicae* (Sulzer) (Hemiptera: aphidae) in organic kale systems of South Texas. Southwestern Entomologist. 40, 1–14. doi: 10.3958/059.040.0101

[B100] Murage A. W. Pittchar J. O. Midega C. A. O. (2015). Gender specific perceptions and adoption of the climate-smart push–pull technology in eastern Africa. Crop Prot. 76, 83–91. doi: 10.1016/j.cropro.2015.06.014

[B101] Murphy-Bokern D. (2022). Developing legume-supported cropping systems in Europe: Have we overlooked something? Ann. Appl. Biol. 181, 133–136. doi: 10.1111/aab.12764

[B102] Mutyambai D. M. Bruce T. J. van den Berg J. Midega C. A. Pickett J. A. Khan Z. R. (2016). An indirect defence trait mediated through igg-induced maize volatiles from neighbouring plants. PloS One 11, 0158744. doi: 10.1371/journal.pone.0158744 PMC4938388 27392034

[B103] Mweke A. Akutse K. S. Ulrichs C. (2020). Integrated Management of *Aphis craccivora* in Cowpea Using Intercropping and Entomopathogenic Fungi under Field Conditions. J. Fungi. 6, 60. doi: 10.3390/jof6020060 PMC7344541 32403358

[B104] Neri F. Mari M. Brigati S. Bertolini P. (2007). Fungicidal activity of plant volatile compounds for controlling *Monilinia laxa* in stone fruit. Plant Dis. 91, 30–35. doi: 10.1094/PD-91-0030 30781062

[B105] Niederbacher B. Winkler J. B. Schnitzler J. P. (2015). Volatile organic compounds as non-invasive markers for plant phenotyping. J. Exp. Bot. 66, 5403–5416. doi: 10.1093/jxb/erv21 25969554

[B106] Nottingham S. Hardie J. Dawson G. Hick A. Pickett J. Wadhams L.. (1991). Behavioral and electrophysiological responses of aphids to host and nonhost plant volatiles. J. Chem. Ecol. 17, 1231–1242. doi: 10.1007/BF01402946 24259180

[B107] Oliva J. Navarro S. Barba A. Navarro G. Salinas M. R. (1999). Effect of pesticide residues on the aromatic composition of red wines. J. Agric. Food Chem. 47, 2830–2836. doi: 10.1021/jf9813135 10552572

[B108] Olufemi O. Pitan R. Odebiyi J. A. (2001). The effect of intercropping with maize on the level of infestation and damage by pod-sucking bugs in cowpea. Crop Prot. 20, 367–372. doi: 10.1016/S0261-2194(00)00135-6

[B109] Ogenga-Latigo M. W. Ampofo J. Baliddawa C. (1992). Influence of maize row spacing on infestation and damage of intercropped beans by the bean aphid (Aphis fabae scop.). i. incidence of aphids. Agric. Food Sci. 30, 111–121. doi: 10.1016/0378-4290(92)90060-M

[B110] Onge A. Cárcamo H. A. Evenden M. L. (2018). Evaluation of semiochemical-baited traps for monitoring the pea leaf weevil, sitona lineatus (Coleoptera: curculionidae) in field pea crops. Environ. Entomol. 47, 93–106. doi: 10.1093/ee/nvx180 29186376

[B111] Palmgren M. G. Edenbrandt A. K. Vedel S. E. Andersen M. M. Landes X. Østerberg J. T.. (2015). Are we ready for back-to-nature crop breeding? Trends Plant Sci. 20 (3), 155–164. doi: 10.1016/j.tplants.2014.11.003 25529373

[B112] Patel H. K. Gomes E. N. Wu Q. Patel N. Kobayashi D. Y. Wang C.. (2023). Volatile metabolites from new cultivars of catnip and oregano as potential antibacterial and insect repellent agents. Front. Plant Sci. 14, 1124305. doi: 10.3389/fpls.2023.1124305 36909430 PMC9995836

[B113] Pawar P. Baskaran R. K. M. Sharma K. C. Marathe A. (2023). Enhancing biocontrol potential of trichogramma chilonis against borer pests of wheat and chickpea. iScience 26. doi: 10.1016/j.isci.2023.106512 PMC10130915 37123237

[B114] Pérez-Hedo M. Gallego-Giraldo C. Forner-Giner M. Á. Ortells-Fabra R. Urbaneja A. (2024). Plant volatile-triggered defense in citrus against biotic stressors. Front. Plant Sci. 15, 1425364. doi: 10.3389/fpls.2024.1425364 39049855 PMC11266131

[B115] Pelzer E. Bazot M. Makowski D. (2012). Pea–wheat intercrops in low-input conditions combine high economic performances and low environmental impacts. Eur. J. Agron. 40, 39–53. doi: 10.1016/j.eja.2012.01.010

[B116] Pickett J. A. Khan Z. R. (2016). Plant volatile-mediated signalling and its application in agriculture: successes and challenges. New Phytologist. 212, 856–870. doi: 10.1111/nph.14274 27874990

[B117] Piechulla B. Lemfack M. C. Kai M. (2017). Effects of discrete bioactive microbial volatiles on plants and fungi. In Plant Cell Environ. 40: 10, 2042–2067. doi: 10.1111/pce.v40.10 28643880

[B118] Pierre J. F. Jacobsen K. L. Latournerie-Moreno L. (2023). A review of the impact of maize-legume intercrops on the diversity and abundance of entomophagous and phytophagous insects. Peer J. 11, e15640. doi: 10.7717/peerj 37397027 PMC10309049

[B119] Piesik A. W. (2011). Volatile Organic Compound Emissions by Winter Wheat Plants (*Triticum aestivum* L.) under Fusarium spp. Infestation and Various Abiotic Conditions. Pol. J. Environ. Stud. 20, 1335–1342.

[B120] Plaszkó T. Kállai Z. Csoma H. Vasas G. Gonda S. (2020). Volatile organic compounds (VOCs) of endophytic fungi growing on extracts of the host, horseradish (*Armoracia rusticana*). Metabolites 10, 451. doi: 10.3390/metabo10110451 33171636 PMC7695154

[B121] Punja Z. K. Utkhede R. S. (2003). Using fungi and yeasts to manage vegetable crop diseases. Trends Biotechnol. 21, 400–407. doi: 10.1016/S0167-7799(03)00193-8 12948673

[B122] Quintana-Rodriguez E. Morales-Vargas A. T. Molina-Torres J. (2015). Plant volatiles cause direct, induced and associational resistance in common bean to the fungal pathogen *Colletotrichum lindemuthianum* . J. Ecol. 103, 250–260. doi: 10.1111/1365-2745.12340

[B123] Rajendran S. Sriranjini V. (2007). Use of fumigation for managing grain quality. Stewart Postharvest Review. 3 (6), 1–8. doi: 10.2212/spr.2007.6.9

[B124] Reddy G. V. P. Sharma A. Gadi R. L. (2018). Biology, ecology, and management of the pea weevil (Coleoptera: chrysomelidae). Ann. Entomol. Soc. Am. 111, 161–171. doi: 10.1093/aesa/sax078

[B125] Reisenman C. E. Lei H. Guerenstein P. G. (2016). Neuroethology of olfactory-guided behavior and its potential application in the control of harmful insects. Front. Physiol. 7, 271. doi: 10.3389/fphys.2016.00271 27445858 PMC4928593

[B126] Rowen E. Kaplan I. (2016). Eco-evolutionary factors drive induced plant volatiles: a meta-analysis. New Phytologist. 210, 284–294. doi: 10.1111/nph.13804 26725245

[B127] Russo A. Pollastri S. Ruocco M. Monti M. M. Loreto F. (2022). Volatile Organic Compounds in the interaction between plants and beneficial microorganisms. Plant Interact. 17, 840–852. doi: 10.1080/17429145.2022.2107243

[B128] Sabraoui A. Lhaloui S. Bouchelta A. El Fakhouri K. El Bouhssini M. (2019). Grain yield losses due to leaf miner (*Liriomyza cicerina* R.) in winter- and spring-planted chickpea in Morocco. Crop Prot. 117, 115–120. doi: 10.1016/j.cropro.2018.11.021

[B129] Sakurai Y. Ishizaki S. Izumi S. Yoshida T. Shiojiri K. Takabayashi J. (2023). The exposure of field-grown maize seedlings to weed volatiles affects their growth and seed quality. Front. Plant Sci. 14. doi: 10.3389/fpls.2023.1141338 PMC10464949 37649992

[B130] Sampaio A. M. Araújo S. S. Rubiales D. Vaz Patto M. C. (2020). Fusarium wilt management in legume crops. Agronomy. 10, 1073. doi: 10.3390/agronomy10081073

[B131] Santos D. R. C. Peñaflor M. F. G. V. Sanches P. A. Nardi C. Bento J. M. S. (2015). The effects of *Gibberella zeae*, Barley Yellow Dwarf Virus, and co-infection on *Rhopalosiphum padi* olfactory preference and performance. Phytoparasitica. 44, 47–54. doi: 10.1007/s12600-015-0493-y

[B132] Sarkar S. H. Wang E. Wu S. (2018). Application of trap cropping as companion plants for the management of agricultural pests: A review. Insects 9, 128. doi: 10.3390/insects9040128 30257517 PMC6316212

[B133] Savelievaa E. I. Gustylevaa L. K. Kessenikha E. D. Khlebnikovaa N. S. Leffingwellb J. Gavrilovac O. P.. (2016). Study of the vapor phase over fusarium fungi cultured on various substrates. Chem. Biodiversity. 13, 891–903. doi: 10.1002/cbdv.201500284 27253722

[B134] Schenkel D. Lemfack M. C. Piechulla B. (2015). A meta-analysis approach for assessing the diversity and specificity of belowground root and microbial volatiles. Front. Plant Sci. 6. doi: 10.3389/fpls.2015.00707 PMC4568395 26442022

[B135] Schenkel D. Maciá-Vicente J. G. Bissell A. Splivallo R. (2018). Fungi indirectly affect plant root architecture by modulating soil volatile organic compounds. Front. Microbiol. Sec. Plant Pathogen Interact. 9. doi: 10.3389/fmicb.2018.01847 PMC6099090 30150975

[B136] Schulz-Bohm K. Zweers H. de Boer W. Garbeva P. (2015). A fragrant neighborhood: Volatile mediated bacterial interactions in soil. Front. Microbiol. 6, 1212. doi: 10.3389/fmicb.2015.01212 26579111 PMC4631045

[B137] Schwartzberg E. G. Böröczky K. Tumlinson J. H. (2011). Pea aphids, *acyrthosiphon pisum*, suppress induced plant volatiles in broad bean, vicia faba. J. Chem. Ecol. 37, 1055–1062. doi: 10.1007/s10886-011-0006-5 21870158

[B138] Semba R. D. Ramsing R. Rahman N. (2021). Legumes as a sustainable source of protein in human diets. Global Food Security. 28, 100520. doi: 10.1016/j.gfs.2021.100520

[B139] Sharma H. C. Manuele T. El Bouhssini M. (2016). “Pest Management in Grain Legumes: Potential and Limitations,” in Integrated Pest Management in the Tropics. Ed. Abrol D. P. (New India Publishing Agency, New Delhi (India), 275–292.

[B140] Shrivastava G. Rogers M. Wszelaki A. (2010). Plant volatiles-based insect pest management in organic farming. Crit. Rev. Plant Sci. 29, 123–133. doi: 10.1080/07352681003617483

[B141] Singh R. Kumar K. Purayannur K. (2022). *Ascochyta rabiei*: A threat to global chickpea production. Mol. Plant Pathol. 23, 1241–1261. doi: 10.1111/mpp.13235 35778851 PMC9366070

[B142] Singh K. D. Mobolade A. J. Bharali R. (2021). Main plant volatiles as stored grain pest management approach: A review. J. Agric. Food Res. 4, 100127. doi: 10.1016/j.jafr.2021.100127

[B143] Soltani A. Ben Abda M. Amri M. Carapelli A. Ben Jemâa J. M. (2020). Seasonal incidence of the leaf miner *Liriomyza cicerina* Rond (Diptera: Agromyzidae) in chickpea fields and effects of climatic parameters, chickpea variety, and planting date on the leaf miner infestation rate. Euro-Mediterranean J. Environ. Integration. 5), 58. doi: 10.1007/s41207-020-00198-4

[B144] Song J. Lee G. Jung J. Moon J. K. Kim S. (2022). Effect of soybean volatiles on the behavior of the bean bug. Riptortus pedestris. J. Chem. Ecology. 48, 207–218. doi: 10.1007/s10886-021-01343-1 35006526

[B145] Soto M. J. López-Lara I. M. Geiger O. (2021). Rhizobia volatiles: potential new players in the complex interkingdom signaling with legumes. Front. Plant Sci. 12. doi: 10.3389/fpls.2021.698912 PMC8258405 34239533

[B146] Splivallo R. Valdez N. Kirchhoff N. Ona M. C. Schmidt J. P. Feussner I.. (2012). Intraspecific genotypic variability determines concentrations of key truffle volatiles. New Phytologist. 194, 823–835. doi: 10.1111/j.1469-8137.2012.04077.x 22394027 PMC3470932

[B147] Tatineni S. Hein G. L. (2023). Plant viruses of agricultural importance: current and future perspectives of virus disease management strategies. Phytopathology. 113, 117–141. doi: 10.1094/PHYTO-05-22-0167-RVW 36095333

[B148] Thomas G. Rusman Q. Morrison W. R. III (2023). Deciphering plant-insect-microorganism signals for sustainable crop production. Biomolecules. 13, 997. doi: 10.3390/biom13060997 37371577 PMC10295935

[B149] Thorn R. M. Greenman J. (2012). Microbial volatile compounds in health and disease conditions. J. Breath Res. 6 (2), 024001. doi: 10.1088/1752-7155/6/2/024001 22556190 PMC7106765

[B150] Valencia-Ortiz M. Marzougui A. Zhang C. (2022). Biogenic VOCs emission profiles associated with plant-pest interaction for phenotyping applications. Sensors. 22, 4870. doi: 10.3390/s22134870 35808366 PMC9269240

[B151] Vinale F. Marra R. Scala F. Ghisalberti E. L. Lorito M. Sivasithamparam K. (2006). Major secondary metabolites produced by two commercial Trichoderma strains active against different phytopathogens. Lett. Appl. Microbiol. 43, 143148. doi: 10.1111/j.1472-765X.2006.01939.x 16869896

[B152] Wahbi S. Prin Y. Thioulouse J. (2016). Impact of wheat/faba bean mixed cropping or rotation systems on soil microbial functionalities. Front. Plant Sci. 7. doi: 10.3389/fpls.2016.01364 PMC5023684 27695462

[B153] War A. R. Paulraj M. G. Ahmad T. (2012). Mechanisms of plant defense against insect herbivores. Plant Signaling Behav. 7, 1306–1320. doi: 10.4161/psb.21663 PMC3493419 22895106

[B154] Webster B. Bruce T. Dufour S. (2008). Identification of volatile compounds used in host location by the black bean aphid, *aphis fabae* . J. Chem. Ecol. 34, 1153–1161. doi: 10.1007/s10886-008-9510-7 18584254

[B155] Weisskopf L. Schulz S. Garbeva P. (2021). Microbial volatile organic compounds in intra-kingdom and inter-kingdom interactions. Nat. Rev. Microbiol. 19, 391–404. doi: 10.1038/s41579-020-00508-1 33526910

[B156] Wyatt T. D. (2018). Queen pheromones, colony odors, and better science: a comment on Holman. Behav. Ecol. doi: 10.1093/beheco/ary074

[B157] Xing Z. Liu Y. Cai W. (2017). Efficiency of trichome-based plant defense in phaseolus vulgaris depends on insect behavior, plant ontogeny, and structure. Front. Plant Sci. 7. doi: 10.3389/fpls.2017.02006 PMC5705610 29225609

[B158] Yi H. S. Heil M. Álvarez R. M. Ballhorn D. J. Ryu C. H. M. (2009). Airborne induction and priming of plant defenses against a bacterial pathogen. Plant Physiol. 151, 2152. doi: 10.1104/pp.109.144782 19812184 PMC2785983

[B159] Yigezu Y. A. El-Shater T. Boughlala M. (2019). Legume-based rotations have clear economic advantages over cereal monocropping in dry areas. Agron. Sustain. Dev. 39, 58. doi: 10.1007/s13593-019-0602-2

[B160] Zeringue H. J. Brown R. L. Neucere J. N. Cleveland T. E. (1996). Relation-ships between C6–C12 alkanal and alkenal volatile contents and resistance ofmaize genotypes to Aspergillus flavus and aflatoxin production. J. Agric. Food Chem. 44, 403–407.

[B161] Zhang C. Chen T. Chen W. Sankaran S. (2023). Non-invasive evaluation of Ascochyta blight disease severity in chickpea using field asymmetric ion mobility spectrometry and hyperspectral imaging techniques. Crop Prot. 165, 106163. doi: 10.1016/j.cropro.2022.106163

[B162] Zhao Y. X. Kang L. (2002). The role of plant odours in the *leafminer Liriomyza sativae* (Diptera: Agromyzidae) and its parasitoid *Diglyphus isaea* (Hymenoptera: Eulophidae): Orientation towards the host habitat. Eur. J. Entomol. 99, 445–450. doi: 10.14411/eje.2002.056

[B163] Zhou J. Zhang N. Wang P. (2015). Identification of host-plant volatiles and characterization of two novel general odorant-binding proteins from the legume pod borer, *maruca vitrata* fabricius (Lepidoptera: crambidae). PloS One 10 (10), e0141208. doi: 10.1371/journal.pone.0141208 26517714 PMC4627759

